# Effect of Delta variant on viral burden and vaccine effectiveness against new SARS-CoV-2 infections in the UK

**DOI:** 10.1038/s41591-021-01548-7

**Published:** 2021-10-14

**Authors:** Koen B. Pouwels, Emma Pritchard, Philippa C. Matthews, Nicole Stoesser, David W. Eyre, Karina-Doris Vihta, Thomas House, Jodie Hay, John I. Bell, John N. Newton, Jeremy Farrar, Derrick Crook, Duncan Cook, Emma Rourke, Ruth Studley, Tim E. A. Peto, Ian Diamond, A. Sarah Walker

**Affiliations:** 1 National Institute for Health Research Health Protection Research Unit in Healthcare Associated Infections and Antimicrobial Resistance, University of Oxford, Oxford, UK; 2 Health Economics Research Centre, Nuffield Department of Population Health, University of Oxford, Oxford, UK; 3 Nuffield Department of Medicine, University of Oxford, Oxford, UK; 4 Department of Infectious Diseases and Microbiology, Oxford University Hospitals NHS Foundation Trust, John Radcliffe Hospital, Oxford, UK; 5 National Institute for Health Research Oxford Biomedical Research Centre, University of Oxford, Oxford, UK; 6 Big Data Institute, Nuffield Department of Population Health, University of Oxford, Oxford, UK; 7 Department of Engineering, University of Oxford, Oxford, UK; 8 Department of Mathematics, University of Manchester, Manchester, UK; 9 IBM Research, Hartree Centre, Sci-Tech Daresbury, UK; 10 Glasgow Lighthouse Laboratory, Glasgow, UK; 11 University of Glasgow, Glasgow, UK; 12 Office of the Regius Professor of Medicine, University of Oxford, Oxford, UK; 13 Health Improvement Directorate, Public Health England, London, UK; 14 Wellcome Trust, London, UK; 15 Office for National Statistics, Newport, UK; 16 MRC Clinical Trials Unit at UCL, University College London, London, UK

**Keywords:** Epidemiology, Viral infection

## Abstract

The effectiveness of the BNT162b2 and ChAdOx1 vaccines against new severe acute respiratory syndrome coronavirus 2 (SARS-CoV-2) infections requires continuous re-evaluation, given the increasingly dominant B.1.617.2 (Delta) variant. In this study, we investigated the effectiveness of these vaccines in a large, community-based survey of randomly selected households across the United Kingdom. We found that the effectiveness of BNT162b2 and ChAdOx1 against infections (new polymerase chain reaction (PCR)-positive cases) with symptoms or high viral burden is reduced with the B.1.617.2 variant (absolute difference of 10–13% for BNT162b2 and 16% for ChAdOx1) compared to the B.1.1.7 (Alpha) variant. The effectiveness of two doses remains at least as great as protection afforded by prior natural infection. The dynamics of immunity after second doses differed significantly between BNT162b2 and ChAdOx1, with greater initial effectiveness against new PCR-positive cases but faster declines in protection against high viral burden and symptomatic infection with BNT162b2. There was no evidence that effectiveness varied by dosing interval, but protection was higher in vaccinated individuals after a prior infection and in younger adults. With B.1.617.2, infections occurring after two vaccinations had similar peak viral burden as those in unvaccinated individuals. SARS-CoV-2 vaccination still reduces new infections, but effectiveness and attenuation of peak viral burden are reduced with B.1.617.2.

## Abstract (web summary)

A large, community-based study in the United Kingdom indicates that the effectiveness of BNT162b2 and ChAdOx1 vaccines against SARS-CoV-2 infections with symptoms or high viral burden is reduced with the Delta variant compared to the Alpha variant.

## Main

Multiple studies have assessed the real-world effectiveness of different Coronavirus 2019 (COVID-19) vaccination programs in the general population, in healthcare and other frontline workers and in care home residents^1^. Studies generally showed high effectiveness of the BNT162b2 mRNA vaccine (Pfizer-BioNTech) and the Oxford-AstraZeneca adenovirus vector vaccine, ChAdOx1 nCoV-19 (termed here ChAdOx1), against the Alpha (B.1.1.7) and preceding variants. More limited real-world effectiveness data are available for the mRNA-1273 (Moderna) vaccine^2–4^. Continued emergence of new SARS-CoV-2 variants potentially threatens the success of vaccination programs, particularly as in vitro experiments suggest reduced neutralization activity of vaccine-elicited antibodies against emerging variants^5,6^. Of particular concern is the Delta variant (B.1.617.2), which has caused sharp rises in infections in many countries, including some with relatively high vaccination coverage, such as the United Kingdom (UK). In England, B.1.617.2 quickly became dominant after being classified as a variant of concern on 28 April 2021, reaching 61% of sequenced positives from the English symptomatic testing program in the week commencing on 17 May (https://assets.publishing.service.gov.uk/government/uploads/system/uploads/attachment_data/file/991343/Variants_of_Concern_VOC_Technical_Briefing_14.pdf) and 99% from 27 June onwards (https://assets.publishing.service.gov.uk/government/uploads/system/uploads/attachment_data/file/1001358/Variants_of_Concern_VOC_Technical_Briefing_18.pdf).

Real-world data on vaccine effectiveness (VE) against B.1.617.2 infections are currently limited. A test-negative case–control study using data to 16 May 2021 from the English symptomatic testing program suggested that the effectiveness after one BNT162b2 or ChAdOx1 vaccination was lower against symptomatic infection with B.1.617.2 (31%) than B.1.1.7 (49%)^7^. Reductions in effectiveness against infection with B.1.617.2 versus B.1.1.7 were smaller after two doses of either vaccine. However, estimates from test-negative case–control studies might be biased if vaccination status influences test-seeking behavior of cases not requiring healthcare^8^. A recent study from Scotland also suggested reduced effectiveness against infection with B.1.617.2 versus B.1.1.7 after two doses of either vaccine^9^. However, the authors found no evidence that effectiveness on hospital admissions in individuals first testing positive varied with B.1.617.2 versus B.1.1.7, leaving it unclear to what extent the results for infection might be attributable to bias due to test-seeking behavior being influenced by vaccination status^8^. A further contributor might be waning immunity, with two recent studies from Israel finding higher infection rates in those vaccinated earliest^10,11^.

We, therefore, assessed the effectiveness of the BNT162b2, ChAdOx1 and mRNA-1273 vaccines against new SARS-CoV-2 PCR-positive cases using the Office for National Statistics (ONS) COVID-19 Infection Survey (CIS), a large, community-based survey of individuals living in randomly selected private households across the UK, where RT–PCR tests were performed after a pre-determined schedule, irrespective of symptoms, vaccination and prior infection^12,13^. Besides avoiding bias from test-seeking behavior changing after receipt of particular vaccines, other advantages over existing studies^7–10,14,15^ include the ability to adjust for prior infection status and a wider range of potential confounders, including working in patient-facing healthcare, care homes or social care, household characteristics and (in)direct contact with hospitals or care homes.

**Extended Data Fig. 1:**
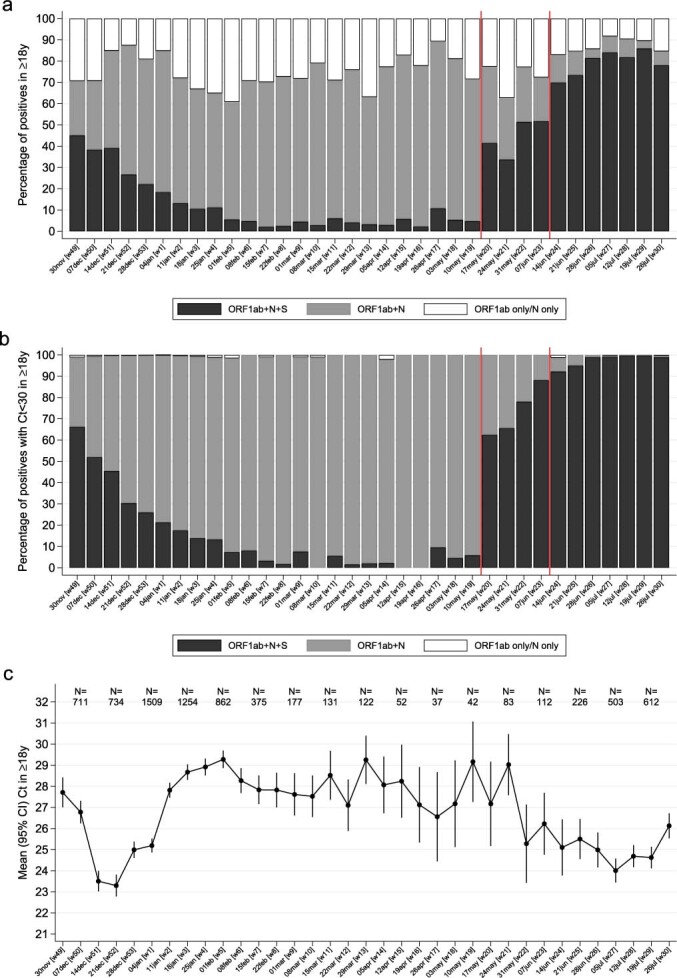
Characteristics of new PCR positive episodes over time. **(a)** gene positivity pattern overall, **(b)** gene positivity pattern restricted to episodes with cycle threshold (Ct) <30; **(c)** and mean Ct value and 95% CI in all positives. Note: analysis among those ≥18 years; ORF1ab + N + S (black) are compatible with wild-type and B.1.617.2 variants (S-gene positive); ORF1ab + N (gray) are compatible with the B.1.1.7 variant (S-gene negative). Those PCR-positives where only a single gene (N or ORF1ab were detected) cannot be classified (vast majority Ct>30). The percentage of PCR-positives with Ct<30 that were ORF1ab + N + S, compatible with B.1.617.2, increased from 6% the week commencing 10 May 2021, to 67% and 92% the weeks starting 17 May and 14 June 2021, respectively. For panel **(c)**, the number of new PCR positive tests in each calendar week are denoted at the top of the graph.

We assessed VE based on overall RT–PCR positivity and split according to self-reported symptoms, cycle threshold (Ct) value (<30 versus ≥30) as a surrogate for viral load, from 1 December 2020 (start of vaccination rollout) to 16 May 2021, when B.1.1.7 dominated, and from 17 May 2021 to 1 August 2021, when B.1.1.7 was replaced by B.1.617.2 (Extended Data Fig. 1), using calendar time as an instrumental variable for variant. In addition, in this B.1.617.2-dominant period, we investigated variation in vaccine effectiveness by time from second vaccination, long-term health conditions, age and prior infection. Given concerns that recent reduced effectiveness of BNT162b2 against (severe) infection in Israel could be due to the short interval between first and second vaccinations (vast majority, 3 weeks^16^), we also investigated the dosing interval for BNT162b2. In addition, we assessed viral burden in new PCR-positive cases occurring ≥14 d after second vaccination using Ct values.

## Results

### Visits and new PCR-positive cases included in analysis

During the B.1.1.7-dominant period, from 1 December 2020 to 16 May 2021 (Extended Data Fig. 1), nose and throat RT–PCR results were obtained from 384,543 individuals aged 18 years or older (221,909 households) at 2,580,021 visits (median (interquartile range (IQR)) 7 (6–8)), of which 16,538 (0.6%) were the first PCR-positive cases in a new infection episode. During the B.1.617.2-dominant period, from 17 May to 1 August 2021, results were obtained from 358,983 individuals (213,825 households) at 811,624 visits (median (IQR) 2 (2,3), 3,123 (0.4%)) being the first PCR-positive cases. Characteristics at included visits are shown in Supplementary Table 1.

**Extended Data Fig. 2:**
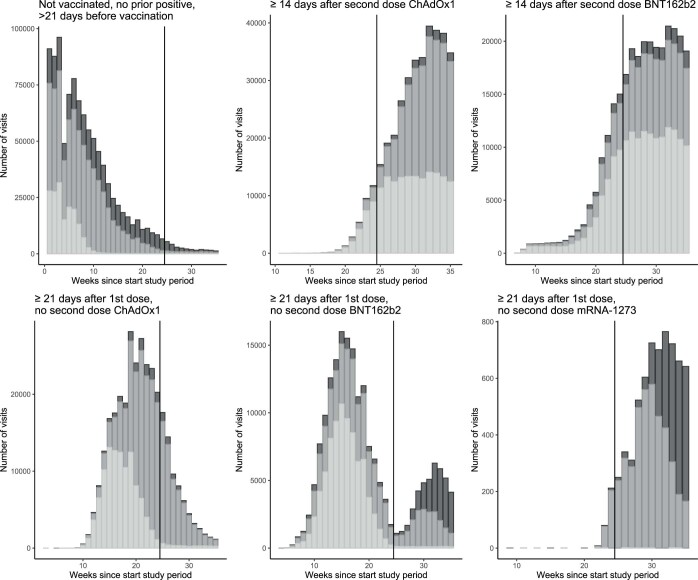
Visits included in analysis over time by vaccination status. The graphs (with different scales for the axes) show the number of visits by vaccination status, by calendar time and age category (dark: 18-34 year olds, intermediate: 35-64 year olds; light: 65+ year olds. The vertical line at 25 weeks indicates the start of the period dominated by B.1.617.2.

**Extended Data Fig. 3:**
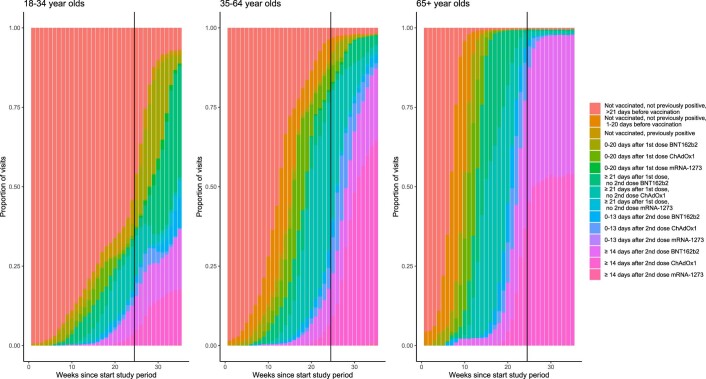
Proportion of visits by exposure. The vertical line at 25 weeks indicates the start of the period dominated by B.1.617.2.

We classified each visit according to vaccination status and prior infection, as previously reported^13^ (Supplementary Table 2), considering individuals not yet vaccinated or >21 d before vaccination without evidence of prior infection as the reference group. The vast majority of post-vaccination visits were with individuals who received BNT162b2 or ChAdOx1; there were only sufficient data to provide conclusive estimates after the first mRNA-1273 dose (Extended Data Figs. 2 and 3 and Supplementary Table 3). The median (IQR) time since first vaccination for visits ≥21 d after the first vaccination but before the second was 47 (34–61), 43 (31–58) and 41 (31–52) for ChAdOx1, BNT162b2 and mRNA-1273, respectively (taking 21 d as the time when protection from the first vaccination might be reasonably achieved^17^). The median (IQR) time from second vaccination for visits ≥14 d after the second vaccination was 41 (27–57) d for ChAdOx1 and 59 (35–86) d for BNT162b2, respectively (taking 14 d as the time when protection from the second vaccination might be reasonably achieved). The median (IQR) dosing interval between first and second vaccination was 76 (68–78) d and 74 (62–77) d, respectively.

### Effect of vaccination on new PCR-positive cases

Adjusting for multiple potential confounders (details in Supplementary Table 1), in the B.1.1.7-dominant period the VE of both BNT162b2 and ChAdOx1 vaccines against new PCR-positive cases was similar in individuals ≥18 years of age to that previously reported to 8 May 2021 in individuals ≥16 years of age^13^ (Table 1 and Supplementary Table 4).

**Table 1:** Effectiveness in individuals older than 18 years of age in B.1.1.7- and B.1.617.2-dominant periods

	BNT162b2: one dose ≥21 d	ChAdOx1: one dose ≥21 d	BNT162b2: second dose 0–13 d ago	ChAdOx1: second dose 0–13 d ago	BNT162b2: second dose ≥14 d	ChAdOx1: second dose ≥14 d	Not vaccinated, previously positive^a^
**VE: all infections**							
1 December 2020–16 May 2021 (B.1.1.7)	59% (52–65%)	63% (55–69%)	77% (66–84%)	72% (50–84%)	78% (68–84%)	79% (56–90%)	60% (50–68%)
17 May 2021 (B.1.617.2)	57% (50–63%)	46% (35–55%)	82% (75–87%)	71% (64–77%)	80% (77–83%)	67% (62–71%)	72% (58–82%)
Heterogeneity *P*	0.60	0.004	0.29	0.99	0.50	0.23	0.12
**VE: Ct** <**30**							
1 December 2020–16 May 2021 (B.1.1.7)	70% (65–74%)	74% (69–79%)	83% (75–89%)	79% (62–88%)	94% (91–96%)	86% (71–93%)	87% (84–90%)
17 May 2021 (B.1.617.2)	62% (56–68%)	50% (41–59%)	81% (73–86%)	69% (61–76%)	84% (82–86%)	70% (65–73%)	77% (66–85%)
Heterogeneity *P*	0.04	<0.0001	0.57	0.25	<0.0001	0.04	0.02
**VE: self-reported symptoms**							
1 December 2020–16 May 2021 (B.1.1.7)	73% (68–76%)	73% (67–77%)	92% (88–95%)	84% (72–91%)	97% (96–98%)	97% (93–98%)	80% (75–84%)
17 May 2021 (B.1.617.2)	58% (51–64%)	40% (28–50%)	93% (90–95%)	73% (66–79%)	84% (82–86%)	71% (66–74%)	82% (73–88%)
Heterogeneity *P*	<0.0001	<0.0001	0.71	0.08	<0.0001	<0.0001	0.59

^a^Re-infection will be a variable amount of time previously, but it was not possible to split this owing to low numbers.

Note: All estimates (VE = 100% × (1 odds ratio)) were obtained from a generalized linear model with a logit link comparing to the reference category of ‘Not vaccinated, not previously positive and ≥21 d before vaccination’ and using clustered robust standard errors. Heterogeneity *P* values were obtained using the two-sided Wald test without adjustment for multiple comparisons. Calendar time was split into two epochs when most cases detected in the survey were ORF1ab + N positive (B.1.1.7 compatible) and then when triple positives became dominant (B.1.617.2 compatible) (Extended Data Fig. 1). Estimates from the former are similar to those from individuals aged ≥16 years previously published on data to 8 May 2021 but with slightly wider 95% CIs due to splitting time after the second dose at 14 d in this analysis. See Supplementary Table 4 for unadjusted heterogeneity *P* values. VE post-second doses changes over time from vaccination (see Fig. 2 and Extended Data Figs. 4 and 5 for changes in individuals aged 18–64 years), so estimates in this table are an average over follow-up included in this analysis.

**Fig. 1:**
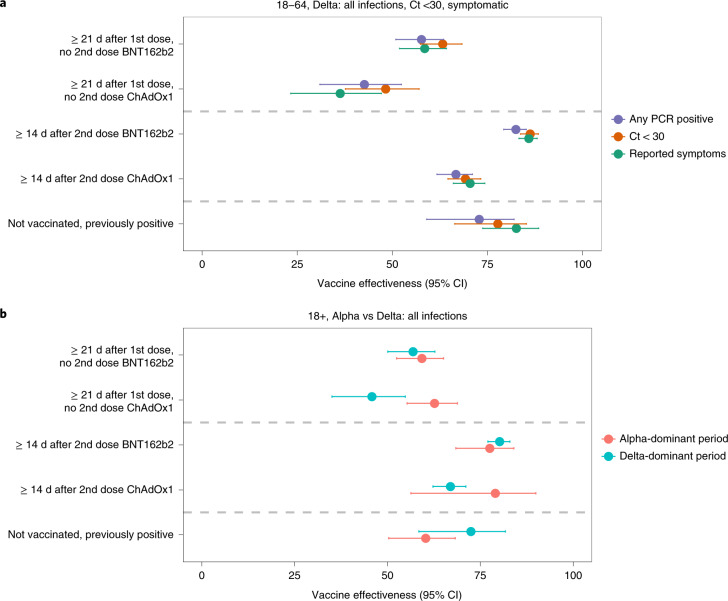
Protection against new PCR-positive cases. **a**, Protection against all new PCR-positive cases, case with Ct < 30, or cases with self-reported symptoms in individuals 18–64 years in the B.1.617.2-dominant period. **b**, Protection against all new PCR-positive cases in individuals older than 18 years in both the B.1.1.7- and B.1.617.2-dominant periods. All estimates (VE = 100% × (1 odds ratio)) were obtained from a generalized linear model with a logit link comparing to the reference category of ‘Not vaccinated, not previously positive and ≥21 d before vaccination’ and using clustered robust standard errors. The dots represent the point estimates (central estimate, 100% × (1 odds ratio)), and the error bars represent 95% CIs. Underlying counts are provided in Supplementary Table 2. VE estimates in Tables 1 and 2 for ≥18 years and 18–64 years, respectively.

In the B.1.617.2-dominant period, in individuals aged ≥18 years, there was evidence of reduced effectiveness compared to the B.1.1.7-dominant period ≥21 d after the first ChAdOx1 vaccination but not ≥14 d after the second (heterogeneity *P* = 0.004 and *P* = 0.23, respectively). There was no evidence of reduced effectiveness in the B.1.617.2-dominant period for BNT162b2 against all new PCR-positive cases (heterogeneity *P* = 0.60 and *P* = 0.23, respectively) (Table 1, Fig. 1 and Supplementary Table 4).

However, a decreasing number of visits remained in the unvaccinated reference group over time, particularly for individuals aged 65 years or over (Extended Data Figs. 2 and 3). In particular, in the B.1.617.2-dominant period, less than 1% of visits of individuals aged 65 years or over were in the unvaccinated reference group, making estimates of VE against this group challenging to interpret. Although reasonable numbers of individuals aged 18–64 years remained in the unvaccinated reference group in the B.1.617.2-dominant period, comparisons with the B.1.1.7-dominant period were not possible in this age group owing to low numbers of individuals having received two vaccinations before 17 May 2021; however, VE estimates in the B.1.617.2-dominant period were similar to all adults for both vaccines (Fig. 1, Tables 1 and 2 and Supplementary Table 4). To investigate VE in the B.1.617.2-dominant period further, we, therefore, focused on this younger age group.

**Table 2:** Effectiveness in individuals aged 18–64 years in the B.1.617.2-dominant period

VE (95% CI)	BNT162b2: one dose ≥21 d	ChAdOx1: one dose ≥21 d	BNT162b2: second dose 0–13 d ago	ChAdOx1: second dose 0–13 d ago	BNT162b2: second dose ≥14 d	ChAdOx1: second dose ≥14 d	Not vaccinated, previously positive^a^
All infections (Fig. 1a)	58% (51–63%)	43% (31–52%)	83% (76–88%)	71% (63–77%)	82% (79–85%)	67% (62–71%)	73% (59–82%)
Ct <30 (Fig. 1b)	63% (57–68%)	48% (38–57%)	81% (73–86%)	69% (61–76%)	86% (84–88%)	69% (65–73%)	78% (66–85%)
Self-reported symptoms (Fig. 1c)	59% (52–64%)	36% (23–47%)	93% (90–95%)	72% (65–78%)	86% (83–88%)	70% (66–74%)	83% (74–88%)
Ct ≥30	40% (31–48%)	27% (12–39%)	87% (82–91%)	74% (66–79%)	71% (65–75%)	59% (53–64%)	57% (35–72%)
No self-reported symptoms	55% (48–61%)	50% (40–58%)	58% (41–70%)	66% (57–73%)	74% (69–78%)	57% (51–63%)	51% (26–67%)

^a^Re-infection will be a variable amount of time previously, but it was not possible to split this owing to low numbers.

Note: All estimates (VE = 100% × (1 odds ratio)) as shown in Fig. 1 were obtained from a generalized linear model with a logit link comparing to the reference category of ‘Not vaccinated, not previously positive and ≥21 d before vaccination’ and using clustered robust standard errors. Heterogeneity *P* values were obtained using the two-sided Wald test without adjustment for multiple comparisons. See Supplementary Table 5 for unadjusted heterogeneity *P* values. See Table 1 for estimates in individuals ≥18 years of age in both B.1.1.7-dominant and B.1.617.2-dominant periods. VE post-second doses changes over time from vaccination (Fig. 2 and Extended Data Figs. 4 and 5), so estimates in this table are an average over follow-up included in this analysis.

In the B.1.617.2-dominant period, VE against new PCR-positive cases of individuals aged 18–64 years was significantly lower for ChAdOx1 versus BNT162b2 ≥21 d after one vaccination and ≥14 d after two vaccinations (heterogeneity *P* = 0.001 and *P* < 0.0001, respectively; Table 2 and Supplementary Table 5). For both vaccines, having received two doses ≥14 d previously still provided significantly more protection than one dose ≥21 d previously (*P* < 0.0001). There was no evidence that the effectiveness of two ChAdOx1 vaccinations ≥14 d previously differed from the protection afforded by previous natural infection without vaccination (heterogeneity *P* = 0.33), whereas two BNT162b2 vaccinations afforded greater protection (*P* = 0.04). Results were similar for individuals ≥18 years of age (Table 1). Effectiveness of a single dose of mRNA-1273 in individuals aged 18–64 years was at least as high as a single dose of BNT162b2 or ChAdOx1 (Supplementary Table 3 and Table 2). Apparent greater effectiveness of a single mRNA-1273 dose could potentially be driven by age, as individuals receiving mRNA-1273 were younger on average, and effectiveness appeared greater in younger individuals (Supplementary Table 6). There were insufficient data to estimate VE after a second mRNA-1273 dose (Extended Data Figs. 2 and 3).

### Effect of time from second vaccination and subgroups

**Fig. 2:**
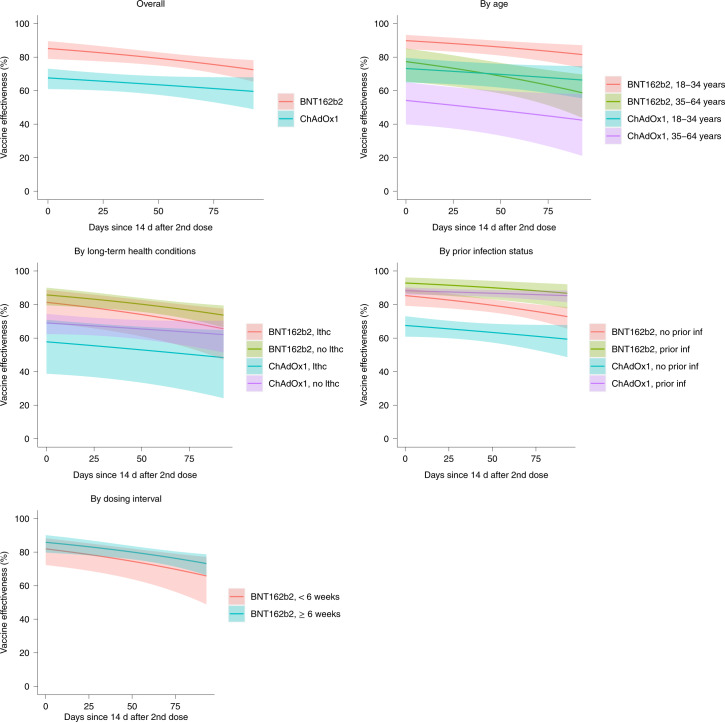
Protection against new PCR-positive cases since second dose. Note: Data were restricted to individuals aged 18–64 years and the B.1.617.2-dominant period. All estimates (VE = 100% × (1 odds ratio)) were obtained from a generalized linear model with a logit link comparing to the reference category of ‘Not vaccinated, not previously positive and ≥21 d before vaccination’ and using clustered robust standard errors. The error bars represent 95% CIs. See Extended Data Figs. 4 and 5 for effects on PCR-positive cases with Ct <30 or symptoms. See Table 3 for estimates of decline. See Supplementary Table 6 for estimates of VE within subgroups 14 d after second vaccination (intercept on panels below). lthc, self-reporting a long-term health condition.

In the B.1.617.2-dominant period, in individuals 18–64 years of age, VE of BNT162b2 against new PCR-positive cases reduced over time (*P* = 0.007; Fig. 2 and Table 3). Reductions were numerically smaller for ChAdOx1, but there was no formal evidence of heterogeneity (*P* = 0.14).

**Table 3:** VE by time from second vaccination

Days since second dose	Effectiveness against any new PCR-positive cases (95% CI)		Effectiveness against Ct <30 (high viral burden) infection (95% CI)		Effectiveness against symptomatic infection (95% CI)	
	BNT162b2	ChAdOx1	BNT162b2	ChAdOx1	BNT162b2	ChAdOx1
14	85% (79–90%)	68% (61–73%)	92% (87–95%)	69% (61–75%)	93% (89–96%)	72% (64–78%)
30	83% (78–88%)	66% (61–71%)	90% (86–93%)	67% (61–73%)	92% (87–95%)	70% (64–76%)
60	80% (76–83%)	64% (58–69%)	85% (81–89%)	65% (58–70%)	86% (82–90%)	67% (60–72%)
90	75% (70–80%)	61% (53–68%)	78% (72–82%)	61% (52–69%)	78% (72–82%)	63% (53–71%)
Relative reduction in effectiveness per month from second dose	22% decline^a^ (6% decline to 41% decline)	7% decline (18% decline to 2% increase)	52% decline^a^ (26% decline to 84% decline)	9% decline (22% decline to 3% increase)	63% decline^a^ (30% decline to 103% decline)	11% decline (26% decline to 2% increase)
Test for evidence of change over time from second dose	*P* = 0.007	*P* = 0.15	*P* < 0.0001	*P* = 0.14	*P* < 0.0001	*P* = 0.10
Test for difference in relative rate of change between the two vaccines	*P* = 0.14		*P* = 0.003		*P* = 0.003	

^a^When initial effectiveness is very high, modest relative declines per month have less effect on absolute effectiveness.

Note: Data are restricted to individuals aged 18–64 years and the B.1.617.2-dominant period. All estimates (VE = 100% × (1 odds ratio)) were obtained from a generalized linear model with a logit link comparing to the reference category of ‘Not vaccinated, not previously positive and ≥21 d before vaccination’ and using clustered robust standard errors. *P* values were obtained using the two-sided Wald test without adjustment for multiple comparisons.

Approximately 10% of visits in the B.1.617.2-dominant period occurred in vaccinated individuals with evidence of prior SARS-CoV-2 infection (Supplementary Table 2). Protection against new PCR-positive cases was significantly higher for vaccinated individuals with prior infection than vaccinated individuals without prior infection for both ChAdOx1 and BNT162b2 (heterogeneity *P* < 0.0001 and *P* = 0.006, respectively; Supplementary Table 6).

VE was also higher in individuals aged 18–34 years than in individuals aged 35–64 years for both ChAdOx1 and BNT162b2 (heterogeneity *P* = 0.002 and *P* = 0.001, respectively). However, there was no evidence of differences between individuals reporting versus not reporting long-term health conditions or between <6 versus ≥6 weeks (median (IQR) 25 (21–34) versus 72 (63–77) d) between the first and second BNT162b2 vaccination (heterogeneity *P* = 0.18; Supplementary Table 6).

### Effect of vaccination by Ct and self-reported symptoms

Restricting new PCR-positive cases to those with Ct <30 (higher viral burden) or with symptoms, attenuations in VE in individuals aged ≥18 years in the B.1.617.2-dominant versus the B.1.1.7-dominant period were more pronounced than against all new PCR-positive cases (Table 1 and Supplementary Table 4). Notably, attenuations in the B.1.617.2-dominant period now reached statistical significance for BNT162b2 as well as ChAdOx1 (for example, heterogeneity *P* < 0.0001 ≥14 d post-second dose for both Ct <30 and symptomatic infections). In the B.1.617.2-dominant period, one or two BNT162b2 vaccinations still provided greater VE than ChAdOx1 against PCR-positive cases with Ct <30 or with symptoms in individuals aged ≥18 years (Table 1; *P* < 0.003) and 18–64 years (Fig. 1, Table 2 and Supplementary Table 5; *P* < 0.001). In the B.1.617.2-dominant period, VE against PCR-positive cases with Ct ≥30 (lower viral burden) or without self-reported symptoms was still lower than against PCR-positive cases with Ct <30 or with symptoms for all three vaccines (Table 2).

**Extended Data Fig. 4:**
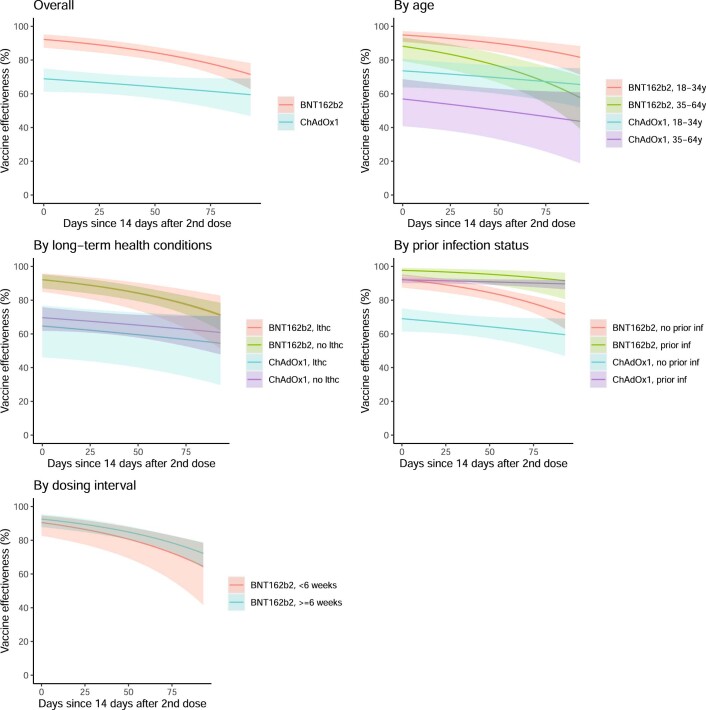
Protection against PCR-positive episodes with Ct < 30. Note: data restricted to those aged 18-64 years old and the B.1.617.2-dominant period; lthc=self-reporting a long term health condition. All estimates (Vaccine effectiveness = 100% * (1-odds ratio)) were obtained from a generalised linear model with a logit link comparing to the reference category of ‘Not vaccinated, not previously positive and ≥21 days before vaccination’ and using clustered robust standard errors. The error bars represent 95% CIs. See Fig. 2 for effects on all PCR-positive episodes. See Table 3 for estimates of overall decline over time. See Supplementary Table 6 for estimates of VE within subgroups 14 days after second vaccination (intercept on panels below).

**Extended Data Fig. 5:**
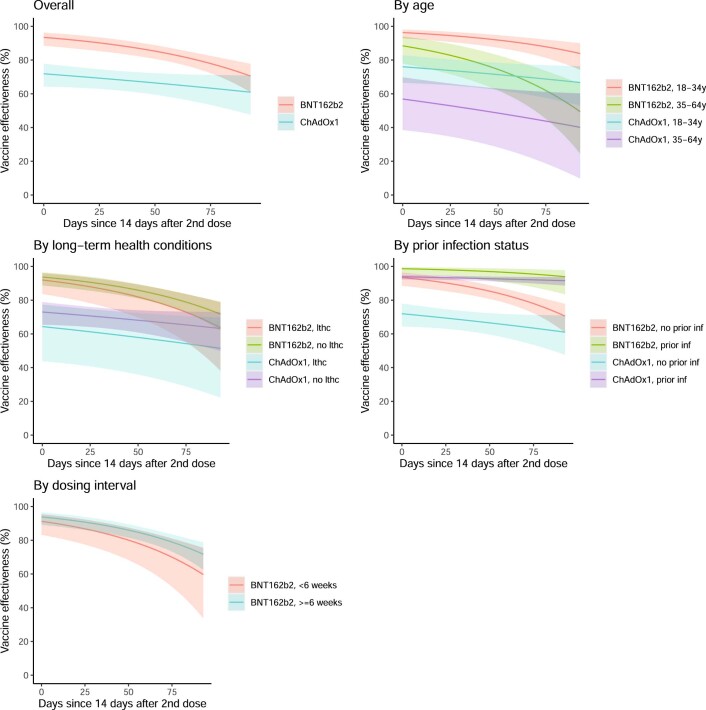
Protection against PCR-positives with reported symptoms. Note: data restricted to those aged 18-64 years old and the B.1.617.2-dominant period; lthc=self-reporting a long term health condition. All estimates (Vaccine effectiveness = 100% * (1-odds ratio)) were obtained from a generalised linear model with a logit link comparing to the reference category of ‘Not vaccinated, not previously positive and ≥21 days before vaccination’ and using clustered robust standard errors. The error bars represent 95% CIs. See Fig. 2 for effects on all PCR-positive episodes. See Table 3 for estimates of overall decline over time. See Supplementary Table 6 for estimates of VE within subgroups 14 days after second vaccination (intercept on panels below).

There was now formal evidence that the effectiveness of BNT162b2 against PCR-positive cases with Ct <30 or with symptoms declined faster ≥14 d after second vaccinations than for ChAdOx1 (heterogeneity *P* = 0.003 for both outcomes; Table 3, and Extended Data Figs. 4 and 5). Extrapolating declines beyond the observed follow-up, both vaccines would be equally effective against PCR-positive cases with Ct <30 139 d (4.6 months) after the second dose and 116 d (3.8 months) against PCR-positive cases with symptoms.

### Viral burden and symptoms in PCR-positive individuals aged ≥18 years

**Fig. 3:**
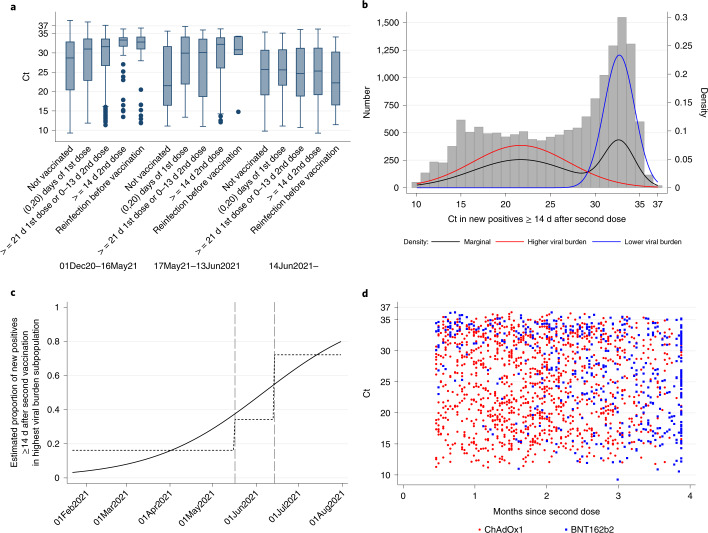
Ct values in new PCR-positive cases in individuals 18 years of age and older. **a**, All new PCR**-**positive cases by vaccination/reinfection status over time (*n* = 15,434). **b**, Distribution of Ct values in all PCR-positive cases ≥14 d after second dose (*n* = 1,736). **c**, Probability that each new PCR-positive case in **b** falls into the higher viral shedding class over time. **d**, Association between Ct values and time from second dose. Note: Boxes in **a** are median (IQR); **b** shows observed Ct values with the marginal density (black) and the densities estimated from a two-component mixture distribution. In **a**, the box plots show median values and upper and lower quartiles of the distribution, with whiskers extending from the hinge to the largest and smallest value no further than 1.5 times the IQR. In **c**, dotted lines show categorical effects of pre-specified calendar periods reflecting B.1.1.7 dominance and early and late B.1.617.2 dominance (Extended Data Fig. 1); the solid line shows a continuous calendar time effect (linear on the log-odds scale). In **d**, months since second dose was truncated at the 95th percentile to avoid the undue influence of outliers. Spearman’s ρ = −0.09 (*P* = 0.004, two-sided *t*-test).

In all 12,287 new PCR-positive cases in the B.1.1.7-dominant period, Ct values (inversely related to viral load) increased strongly with increasing time from first vaccination and number of doses (age/sex-adjusted trend *P*< 0.0001; Fig. 3a and Supplementary Table 7). Ct values were highest in individuals ≥14 d after second vaccination—significantly higher than in individuals who were unvaccinated and not previously PCR/antibody positive but with no evidence that they differed from individuals who were unvaccinated but previously PCR/antibody positive (age/sex-adjusted *P* = 0.02 and *P* = 0.72, respectively).

**Extended Data Fig. 6:**
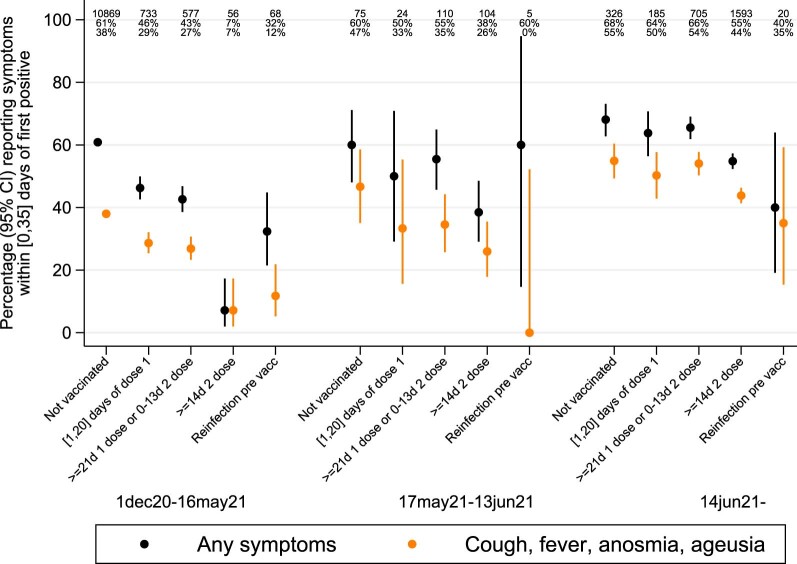
Symptoms reported in PCR-positives by subgroup. Note: data restricted to those 18+ years old. Error bars represent 95% confidence intervals. The number of tests in each group and the corresponding percentages with any symptoms and classic symptoms (cough, fever, anosmia, ageusia) are denoted in the top of the graph.

**Extended Data Fig. 7:**
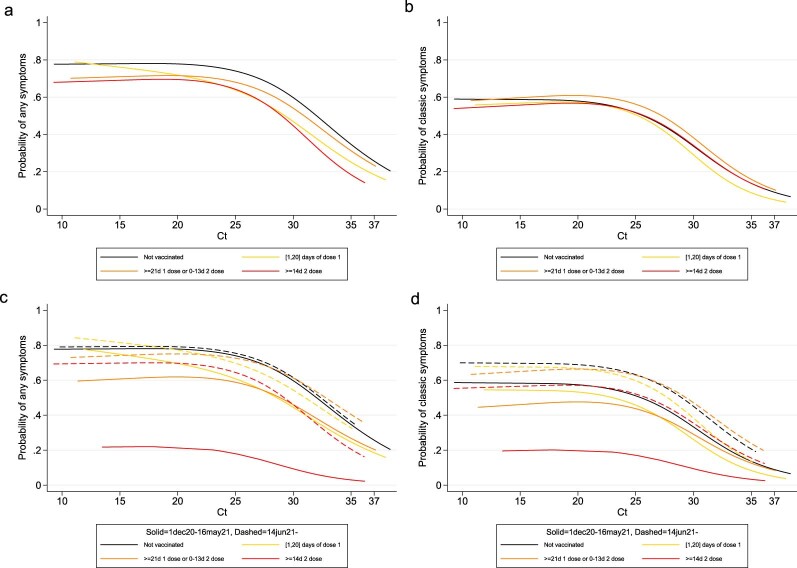
Probability of reporting symptoms in new PCR-positives. Note: data include all new PCR positives among those ≥18 years old. Panels **(a)** and **(c)** relate to any symptoms, while **(b)** and **(d)** relate to classic symptoms (cough, fever, loss of taste/smell). Panels **(a)** and **(b)** include all PCR-positives from 1 December 2020 to 1 August 2021; panels **(c)** and **(d)** fit separate models to the periods 1 December 2020-16 May 2021 (solid lines) and 14 June 2021-1 August 2021 (dashed lines).

From 14 June 2021, after which more than 92% of PCR-positive cases with Ct <30 were B.1.617.2 compatible (Extended Data Fig. 1), differences in Ct values between individuals who were unvaccinated and individuals ≥14 d after second vaccination had attenuated substantially (age/sex-adjusted *P* = 0.35, heterogeneity versus B.1.1.7-dominant period *P* = 0.01), as had differences with individuals who were unvaccinated but previously PCR/antibody positive. Mirroring the attenuation in Ct values, the difference between individuals who were unvaccinated and individuals ≥14 d after second vaccination in the percentages of PCR-positive cases reporting any or well-recognized COVID-19 symptoms (cough, fever or loss of taste/smell) significantly attenuated after 14 June 2021 (heterogeneity *P* < 0.0001 and *P* = 0.008 respectively; Extended Data Fig. 6). However, this was likely driven by lower Ct values, as the association between Ct and symptom reporting remained broadly similar after B.1.617.2 (Extended Data Fig. 7).

Considering all 1,736 PCR-positive cases ≥14 d after two ChAdOx2 or BNT162b2 vaccinations from 1 December 2020 through 1 August 2021 (1,415 (82%), of whom had ≥1 prior negative swabs after their second vaccination), Ct values came from a mixture of two subpopulations (Fig. 3b). The low subpopulation had a mean Ct of 21.7 (95% confidence interval (CI), 21.2–22.2), and the high subpopulation had a mean Ct of 32.7 (95% CI, 32.5–33.0), consistent with either mild or late identified infection. The relative percentage of new PCR-positive cases falling into these two subpopulations varied strongly over time (*P* < 0.0001; Fig. 3c), with the percentage in the low Ct (high viral burden) subpopulation averaging 16%, 34% and 72% through 16 May 2021, 17 May–13 June and 14 June onwards, respectively.

**Extended Data Fig. 8:**
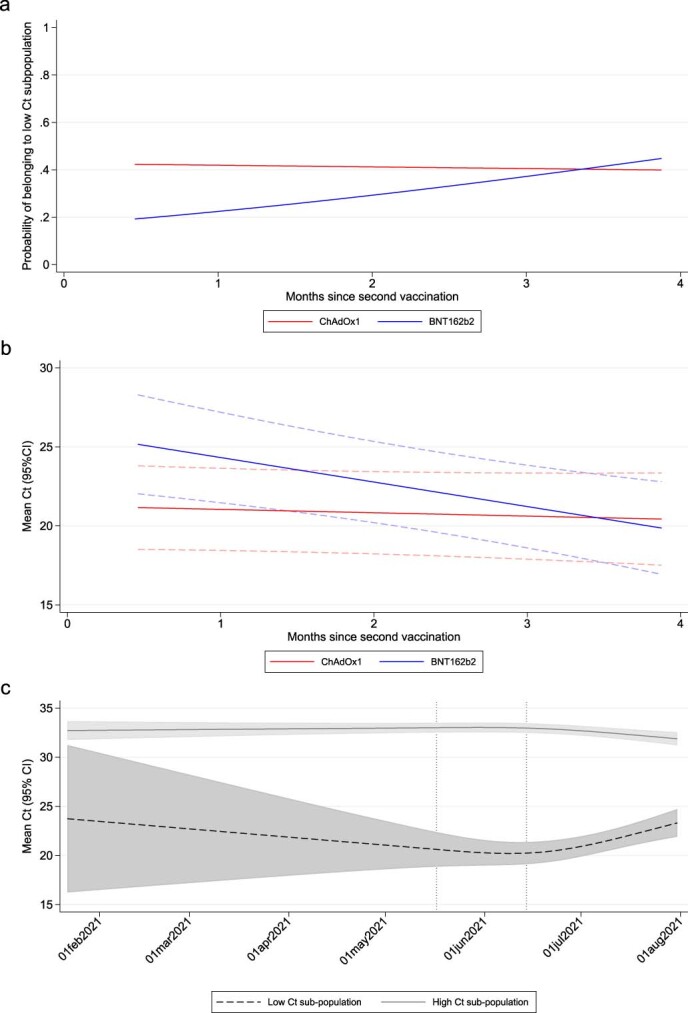
Low Ct populations ≥14 days after second vaccination. **(a)** Adjusted effects of vaccine type and months since second vaccination on probability of belonging to the low Ct sub-population; **(b)** adjusted effects of vaccine type and months since second vaccination on Ct values within the low Ct sub-population**; (c)** adjusted effects of calendar time on Ct values within the low Ct sub-population. Shaded areas represent 95% confidence intervals. Note: data include all new PCR positives among those ≥18 years old; estimated at the reference category for other factors (27 April 2021, male, no previous PCR/antibody-positive, not reporting a long-term health condition). In **(c)**, test for non-linearity in effect of calendar date p = 0.003 for low and <0.0001 for high Ct sub-population (two-sided Wald test without adjustment for multiple comparison).

Independently of this effect of calendar time (reflecting B.1.1.7 versus B.1.617.2 dominance), new PCR-positive cases were less likely to be in the low Ct subpopulation 14 d after two BNT162b2 vaccinations than two ChAdOx1 vaccinations (adjusted odds ratio (aOR) = 0.33 (95% CI, 0.16–0.67), *P* = 0.002), but this likelihood increased significantly over time from second vaccination (aOR per month = 1.43 (95% CI, 1.07–1.91), *P* = 0.01; unadjusted in Fig. 3d; Supplementary Table 7 and Extended Data Fig. 8). In contrast, there was no evidence of changing likelihood over time for ChAdOx1 (aOR per month = 0.97 (95% CI, 0.79–1.19), *P* = 0.78; heterogeneity *P* = 0.02). Overall, therefore, by around 3 months after second vaccination, the probability of being in the low-Ct subpopulation was similar for both BNT162b2 and ChAdOx1. Vaccine type and time from second vaccination had similar effects on the mean Ct within the low-Ct subpopulation, with higher Ct values in new PCR-positive cases 14 d after second BNT162b2 vaccination (*P* = 0.003), which then dropped significantly faster with time from second vaccination than for ChAdOx1 (interaction *P* = 0.01), leading to similar Ct values with both vaccines by around 3 months (Extended Data Fig. 8b). Individuals who were previously PCR/antibody positive were less likely to belong to the low-Ct subpopulation compared to individuals without evidence of previous infection (*P* < 0.0001), while individuals who reported having long-term health conditions were also associated with a lower probability of belonging to the low-Ct subpopulation (*P* = 0.006), potentially reflecting protection in the former and longer duration of PCR positivity in the latter, leading to late infections being more likely to be identified through the fixed testing schedule. There were no additional effects of sex, age (unadjusted in Extended Data Fig. 8b) or ethnicity on the probability of belonging to the low-Ct subpopulation (*P* > 0.15).

**Extended Data Fig. 9:**
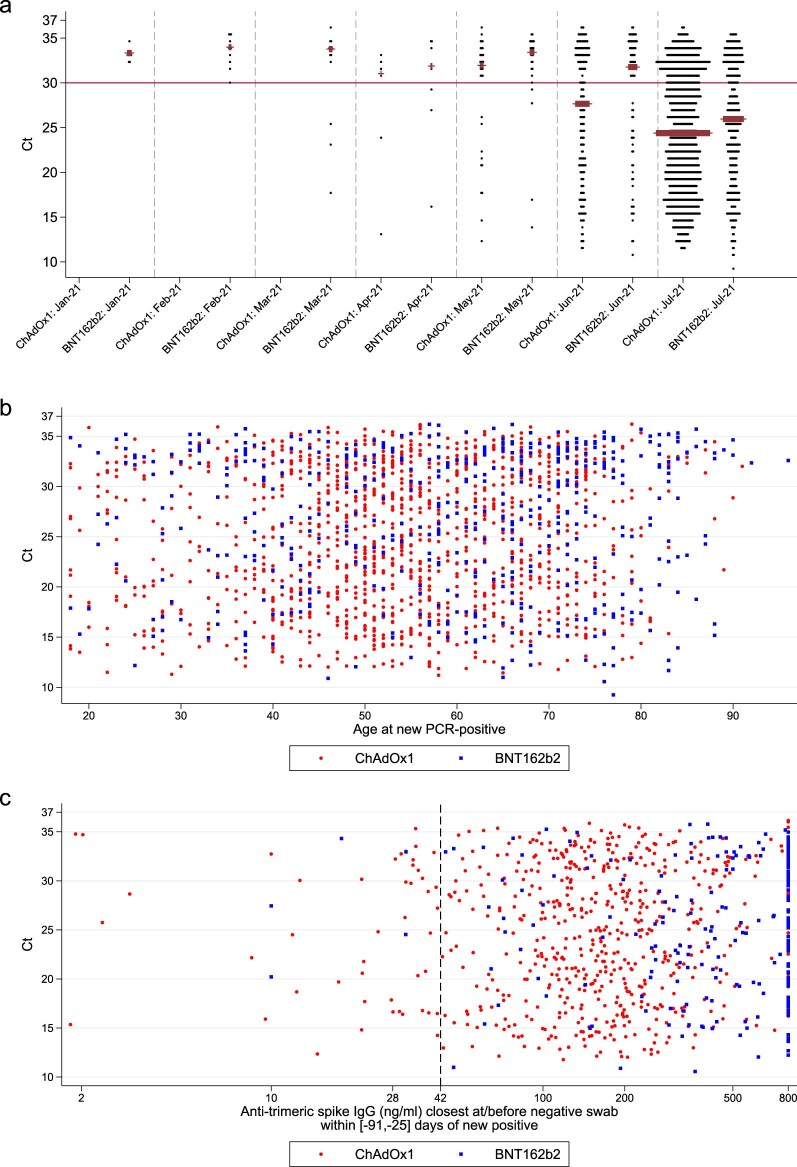
Ct values in new PCR-positives ≥14 days after second vaccination. **(a)** by months since second vaccination (N = 1,736), **(b)** by age (N = 1,736) and **(c)** by most recent anti-trimeric spike IgG antibody measurement where available (N = 846). Note: in **(a)** red solid line shows 30 threshold used in main analysis. Short red lines show median within groups. In **(c)** antibody measurements taken median 30 (IQR 28-54) [range 25–91] days before the new PCR-positive, at or before the most recent prior negative swab and 14 days or more after first vaccination. 42 ng/ml is the positivity threshold. Overall association with Ct Spearman rho=0.08 (p = 0.002) for age **(b)**, and 0.10 (p = 0.002) for IgG **(c)**. P-values for the Spearman tests were obtained using a two-side t-test.

**Extended Data Fig. 10:**
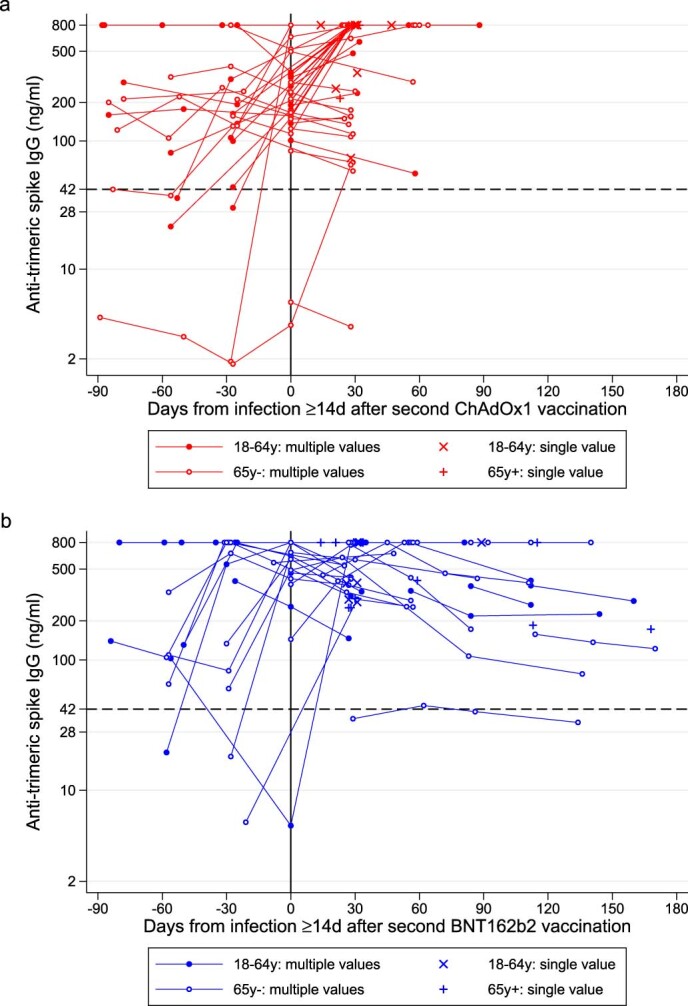
Antibody changes upon PCR-positive ≥14 days after second vaccination. **(a)** ChAdOx1 (N = 60), **(b)** BNT162b2 (N = 51]. Lines join repeated observations from the same individual. Median second vaccination date (IQR) 24 Apr 2021 (15 April-18 May) for ChAdOx1 and 5 April (9 January-16 April 2021) for BNT162b2. Median (IQR) new PCR-positive date 13 June (30 May-19 June) for ChAdOx1 and 25 May 2021 (20 February-16 June) for BNT162b2.

Anti-trimeric spike antibody (IgG) levels were measured in a subset of individuals, selected at random or based on longest study participation or prior swab positivity (Methods). A prior result was available for 846/1,736 (49%) new PCR-positive cases ≥14 d after two ChAdOx2 or BNT162b2 vaccinations, of which 795 (94%) were above the 42 ng ml^−1^ positivity threshold (Extended Data Fig. 9c) (median, 215 ng ml^−1^) (IQR 126–454). However, independently of factors in Supplementary Table 7, every doubling in IgG was associated with 22% lower odds of a new PCR-positive case belonging to the low-Ct subpopulation (aOR = 0.78 (95% CI, 0.66–0.93), *P* = 0.007), with no evidence that this varied by vaccine type (heterogeneity *P* = 0.31). There was no evidence of association between IgG and mean Ct values within either subpopulation (*P* > 0.14). Most individuals with antibody measurements after a new PCR-positive test ≥14 d after second vaccination increased antibody levels after their new PCR-positive test, suggesting a boosting effect of new infections after vaccination (Extended Data Fig. 10).

## Discussion

Our results suggest that vaccination with two doses of BNT162b2 or ChAdOx1 still substantially reduces the risk of new PCR-positive SARS-CoV-2 infections. However, whereas the two vaccines provided similar benefits when B.1.1.7 was dominant, benefits from two ChAdOx1 doses are reduced more with B.1.617.2 than for two BNT162b2 doses, although two ChAdOx1 doses still provide similar protection as that from previous natural infection. Benefits from both vaccines are numerically greater against PCR-positive cases in patients with versus without self-reported symptoms and in patients with high- versus low-viral-burden PCR-positive cases, but the difference in effectiveness is smaller with B.1.617.2 for both vaccines.

The dynamics of protection varied over time from second vaccination and by vaccine type, with initially larger effectiveness with BNT162b2 than ChAdOx1, which then become more similar by ~4–5 months due to more rapid waning of effectiveness with BNT162b2, particularly against infections with Ct <30 or symptoms. Notably, there was no evidence that effectiveness depended on the interval between first and second BNT162b2 vaccinations (<6 weeks versus ≥6 weeks). Protection against new PCR-positive cases was significantly larger in vaccinated individuals with evidence of prior infection than in vaccinated individuals without prior infection.

We also found greater effectiveness in individuals 18–34 years old than individuals 35–64 years old, although we were not able to jointly assess the degree to which this could have been caused by higher rates of previous infection in this group. We were unable to estimate VE in individuals 65 years of age and older in the B.1.617.2-dominant period, as very few individuals remained unvaccinated in the reference group; moreover, such individuals are unlikely to be representative. This challenge of diminishing and increasingly unrepresentative control groups also applies to other designs, such as test-negative case–control, and will increasingly hinder assessment of VE at younger ages with increasing rollout (Extended Data Fig. 3).

Few studies have assessed VE during periods where the B.1.617.2 variant dominated. A test-negative case–control study from the English symptomatic testing program suggested that the effectiveness after one dose of either BNT162b2 or ChAdOx1 was lower against symptomatic infection with B.1.617.2 than B.1.1.7 (31% versus 49%, respectively), with smaller differences after two doses (BNT162b2, 88% versus 94%, respectively; ChAdOx1, 67% versus 75%, respectively)^7^. There is little alternative to using observational data to assess VE against new variants, because additional placebo-controlled randomized trials would be unethical (although active comparator trials could still be performed). However, there are many biases in observational analyses^18^, particularly if symptomatic testing is non-random and related to perceived efficacy^8^. Potential bias due to such health-seeking behavior is likely particularly pronounced for mild symptoms, included in many VE studies using routine symptomatic testing program data. This might be exacerbated by the generic nature of many symptoms prompting testing, which might be incidental, and misclassification due to individuals reporting symptoms when they want to get a test. As we demonstrated substantially lower VE against infections with high Ct or no reported symptoms, this would bias estimates toward lower effects, potentially differentially between vaccines.

Such bias is substantially reduced when testing schedules are fixed independent of symptom or vaccination status, as in our survey, or when using objective severe disease endpoints, such as hospital admissions and deaths. A recent study from Scotland^9^ found no statistical evidence of differential effectiveness against hospital admissions with B.1.617.2 and B.1.1.7 (62% versus 72% in PCR-positive cases), although power was relatively limited. BNT162b2 effectiveness against hospitalizations remained high when B.1.617.2 dominated in Israel (88%, https://www.gov.il/BlobFolder/reports/vaccine-efficacy-safety-follow-up-committee/he/files_publications_corona_two-dose-vaccination-data.pdf), despite lower effectiveness against self-reported symptomatic SARS-CoV-2 infection (41% versus 97% previously)^19^.

Although testing behavior bias could contribute to these differences, we also found a stronger protective effect against infections with higher viral burden and/or symptoms from BNT162b2 and ChAdOx1 vaccines, although to a lesser degree than against B.1.1.7. One explanation could be differential effects of vaccination on mucosal and systemic immunity^20^. In theory, the former is more important for preventing carriage, transmission and infection becoming established, whereas the latter is more important for preventing severe disease once infected^21^. Studies in rhesus macaques showed greater reductions in SARS-CoV-2 viral load in the lungs and prevention of pneumonia, without reducing viral loads in the upper respiratory tract with intramuscular ChAdOx1 (ref. ^22^), and protection against viral replication at much lower concentrations in the lower respiratory tract than in the upper respiratory tract with intramuscular mRNA-1273 (ref. ^23^). In mice, an experimental adenovirus vaccine induced strong systemic adaptive immune responses against SARS-CoV-2 and reduced infection in the lungs but minimal mucosal immune responses when administered intramuscularly^24^. Another explanation for differences in VE against infections with B.1.617.2 versus B.1.1.7 is that the former might have a replication advantage in airway human epithelial cells; increased infectivity at mucosal surfaces could facilitate antibody evasion^25^. A final explanation could be varying protection by time since second vaccination in the B.1.617.2-dominant period, which also differed between BNT162b2 and ChAdOx1. When such time-dependent effects are present, studies with different follow-up will inevitably get different ‘average’ results, and studies when B.1.1.7 dominated might predominantly reflect early effects. Regardless of explanation, although protection against hospitalization and death is maintained, ‘booster’ vaccinations might not be needed, particularly because infection after vaccination might provide a natural antibody boost. However, declines in immunity against infection show that this needs to be monitored closely.

In addition to reduced VE, we found a substantial shift in viral burden in individuals who were infected despite two vaccinations with BNT162b2 or ChAdOx1 in the B.1.617.2-dominant period, with similar average Ct values to individuals infected without vaccination, and much more similar percentages reporting symptoms, driven by Ct. Although, with B.1.1.7, we^13^ and others^26–28^ found that vaccinated individuals had lower viral burden (higher Ct values) than unvaccinated individuals, the greater number of new PCR-positive cases (1,736 ≥14d after second vaccination) allowed us to show that there are two different types of such infections: a low-viral-burden group that dominated early in 2021 and a high-viral-burden group that increased in frequency with B.1.617.2. Individuals receiving ChAdOx1 were more likely to fall into the latter group after their second vaccination, as were an increasing percentage of new PCR-positive individuals with increasing time from second BNT162b2 vaccination, mirroring changes in protection against new PCR positivity. Peak viral load, therefore, now appears similar in infected vaccinated and unvaccinated individuals, with potential implications for onward transmission risk, given the strong association between peak Ct and infectivity^29^. However, the degree to which this might translate into new infections is unclear; a greater percentage of virus might be non-viable in individuals who are vaccinated, and/or their viral loads might also decline faster, as suggested by a recent study of patients hospitalized with B.1.617.2 (ref. ^26^) (supported by associations between higher Ct and higher antibody levels here and in ref. ^30^), leading to shorter periods ‘at risk’ for onwards transmission. Nevertheless, there might be implications for any policies that assume a low risk of onward transmission from vaccinated individuals (for example, relating to self-isolation and travel), despite vaccines both still protecting against infection, thereby still reducing transmission overall. This might be particularly important when vaccinated individuals are not aware of their infection status or perceive that their risk of transmission is low. Notably, individuals infected after second vaccination appeared to gain an antibody boost, and higher prior antibody levels were independently associated with lower viral burden.

The main study strength is its size and design including participants from randomly selected private residential households in the community, tested following a fixed schedule, independent of symptoms and vaccination status, thereby avoiding bias due to test-seeking behavior that potentially affects many other studies assessing VE against SARS-CoV-2 infections^8^. Furthermore, we are able to adjust for risk factors that also affect vaccination but are typically not available in electronic health records, such as patient-facing healthcare work and long-term health conditions, and also adjusted for background ‘force of infection’ using flexible models for background infection rates varying by age, calendar time and geographical region. This should lead to less residual confounding than studies relying on routine electronic healthcare data.

Our study has several limitations. Although we included a broad set of potential confounders, results might still be biased by unknown confounders or misclassification of prior infection status—for example, due to having antibody measurements on only a subset. Participants are tested initially at weekly and then monthly visits, meaning that, when rates are increasing, as when B.1.617.2 came to dominate, we expect to identify infected individuals earlier in their infection episode^31,32^, as shown and adjusted for in our Ct analysis. Late detection of older infections on the fixed visit schedule means that some positives could be classified as having occurred shortly after vaccination, whereas the infection might actually have been acquired before vaccination, potentially diluting VE estimates. However, most infections ≥14 d after second vaccination had a preceding negative after second vaccination. To avoid misclassification bias from erroneously classifying higher Ct positives where only ORF1ab + N genes were detected as B.1.1.7, our comparisons treated calendar periods as an instrumental variable, according to whether B.1.1.7 or B.1.617.2 was dominant, but this will likely lead to a small amount of bias in our VE estimates. In particular, it is expected to result in a small dilution bias when estimating the effect of the B.1.617.2 variant. We did not have information on severe outcomes, against which VE might remain high as hospitalization and death rates have increased by only small amounts in the UK, despite large increases in the number of people testing positive (https://coronavirus.data.gov.uk/).

In summary, with B.1.617.2, BNT162b2 and ChAdOx1 remain protective against any new PCR-positive cases and infections with higher viral burden or symptoms, but VE against these outcomes is reduced, with evidence of significantly different dynamics of immunity against infections with Ct <30 or symptoms after second doses of the two vaccines. With B.1.617.2, those infections occurring despite either vaccine have similar peak viral burden to those in unvaccinated individuals. The effect on infectivity to others is unknown but requires urgent investigation. It further argues for vaccinating as many of the population as possible, because unvaccinated individuals might not be protected by as substantial reductions in transmission among the immunized population as seen other infections, making herd immunity likely unachievable for emerging variants and requiring efforts to protect individuals themselves. Although the current preservation of VE against severe outcomes in other studies suggests that allowing ongoing virus transmission and nasopharyngeal viral presence might have limited consequences, the success of this strategy will ultimately rely on universal vaccination (currently not available to most worldwide); uniform protection induced by vaccines, including in older individuals; optimization of vaccine strategies to induce higher levels of mucosal and systemic immunity; and an absence of novel variants that might compromise VE against severe infection.

## Methods

The survey methods are the same as those described previously^13^ but are also described in detail below.

### Study participants

The ONS CIS is a large household survey with longitudinal follow-up (ISRCTN21086382, https://www.ndm.ox.ac.uk/covid-19/covid-19-infection-survey/protocol-and-information-sheets) (details in refs. ^12,13^). The study received ethical approval from the South Central Berkshire B Research Ethics Committee (20/SC/0195). Private households are randomly selected on a continuous basis from address lists and previous surveys to provide a representative sample across the UK. After verbal agreement to participate, a study worker visited each selected household to take written informed consent for individuals aged 2 years and over. Parents or carers provided consent for those aged 2–15 years; those aged 10–15 years also provided written assent. For the current analysis, we included only individuals aged 16 years and older who were potentially eligible for vaccination.

Individuals were asked about demographics, behaviors, work and vaccination uptake (https://www.ndm.ox.ac.uk/covid-19/covid-19-infection-survey/case-record-forms). At the first visit, participants were asked for (optional) consent for follow-up visits every week for the next month and then monthly for 12 months from enrollment. At each visit, enrolled household members provided a nose and throat self-swab following instructions from the study worker. From a random 10–20% of households, individuals age 16 years or older were invited to provide blood monthly for antibody testing from enrollment. From April 2021, additional participants were invited to provide blood samples monthly to assess vaccine responses, targeting 150,000 antibody tests per month, based on a combination of random selection and prioritization of individuals in the study for the longest period (independent of test results, vaccination or previous positive PCR tests). Throughout, individuals with a positive swab test and their household members were also invited to provide blood monthly for follow-up visits after this.

### Laboratory testing

Swabs were couriered directly to the UK’s national Lighthouse laboratories (Glasgow and the National Biocentre in Milton Keynes (to 8 February 2021)) where samples were tested within the national testing program using identical methodology. The presence of three SARS-CoV-2 genes (ORF1ab, nucleocapsid protein (N) and spike protein (S)) was identified using RT–PCR with the TaqPath RT–PCR COVID-19 kit (Thermo Fisher Scientific), analyzed using UgenTec FastFinder 3.300.5 (TagMan 2019-nCoV assay kit V2 UK NHS ABI 7500 v2.1, UgenTec). The assay plugin contains an assay-specific algorithm and decision mechanism that allows conversion of the qualitative amplification assay raw data into test results with little manual intervention. Samples are called positive if either N or ORF1ab, or both, are detected. The S gene alone is not considered a reliable positive but could accompany other genes (that is, one, two or three gene positives).

Blood samples were couriered directly to the University of Oxford where they were tested for the SARS-CoV-2 antibody using an ELISA detecting anti-trimeric spike IgG^33^. Before 26 February 2021, the assay used fluorescence detection (positivity threshold, 8 million units)^33^. After this, it used a commercialized CE-marked version of the assay—the Thermo Fisher OmniPATH 384 Combi SARS-CoV-2 IgG ELISA (Thermo Fisher Scientific)—with the same antigen and a colorimetric detection system (positivity threshold, 42 ng ml^−1^ monoclonal antibody unit equivalents, determined from 3,840 samples run in parallel). From 27 February 2021, samples were also tested using a Thermo Fisher Scientific N antibody.

### Inclusion and exclusion criteria

This analysis included individuals aged 18 years or older (that is, those who were eligible for vaccination) and all visits with positive or negative swab results from 1 December 2020 to 1 August 2021. The analysis of VE comparing B.1.1.7-dominant and B.1.617.2-dominant periods included all individuals aged ≥18 years; analyses of the B.1.617.2-dominant period were also restricted to visits in individuals aged 18–64 years, as the vast majority of individuals 65 years and older were vaccinated twice before B.1.617.2 became dominant (Extended Data Figs. 2 and 3). Analyses of Ct values in new PCR-positive cases by vaccination status included all individuals aged ≥18 years.

### Vaccination status

Individuals were asked about their vaccination status at visits, including type, number of doses and date(s). Individuals from England were also linked to administrative records from the National Immunisation Management Service (NIMS). We used records from NIMS where available; otherwise, we used records from the survey, because linkage was periodic, and NIMS does not contain information about vaccinations received abroad or in Northern Ireland, Scotland and Wales. Where records were available in both, agreement on type was 98%, and agreement on dates was 95% within ±7 d. A small number of visits after reported vaccination with either unknown or vaccines other than ChAdOx1, BNT162b2 or mRNA-1273 were excluded as these were too few to provide reliable estimates (for mRNA-1273, we included only the first dose and the period ≥17 May because numbers were also too few before 17 May and for second doses (Extended Data Fig. 3)).

### SARS-CoV-2-positive cases

PCR-positive results might be obtained at multiple visits after infection, so we grouped positive tests into episodes (cases). Whole genome sequencing is available on only a subset of positives, and only a subsample provides monthly blood samples for antibody status, so positive episodes were defined using study PCR results. We previously found that defining episodes based on 90 d, as suggested by the World Health Organisation (https://www.paho.org/en/documents/interim-guidelines-detecting-cases-reinfection-sars-cov-2), led to higher than plausible risk of a new episode between 90 and 120 d, particularly for high-Ct infections^13^, suggesting that intermittent long-term PCR positivity could be contributing. Here, we, therefore, defined the start of a new ‘positive case’ as the date of (1) the first PCR-positive test in the study (not preceded by any study PCR-positive test by definition); (2) a PCR-positive test after four or more consecutive negative tests; or (3) a PCR-positive test at least 120 d after the start of a previous episode with one or more negative tests immediately preceding this. Positive cases were used to classify exposure groups and outcomes (see below).

### Exposures

At each study visit, a participant was classified into one of 13 different exposure groups based on current vaccination status, study antibody and PCR tests and (for exposure classification only) positive swab tests linked from the English national testing program (https://www.gov.uk/government/publications/nhs-test-and-trace-statistics-england-methodology/nhs-test-and-trace-statistics-england-methodology) (before visit), as follows:

i. Visits from participants ≥21 d before first vaccination, including those currently with no vaccination date, with no prior PCR- or antibody-positive episode in the study, nor a positive swab test in the national testing program (as defined below) (‘Not vaccinated, not previously positive, ≥21 d before vaccination’) (baseline group);
ii. Visits from participants 1–21 d before first vaccination with no prior PCR- or antibody-positive episode in the study, nor a positive swab test in the national testing program (‘Not vaccinated, not previously positive, 1–21 d before vaccination’)
iii. Visits 0–20 d after a first vaccination with BNT162b2 (‘Vaccinated 0–20 d ago BNT162b2’);
iv. Visits 0–20 d after a first vaccination with ChAdOx1 (‘Vaccinated 0–20 d ago ChAdOx1’);
v. Visits 0–20 d after a first vaccination with mRNA-1273 (‘Vaccinated 0–20 d ago mRNA-1273’);
vi. Visits 21 d or more after a first vaccination with BNT162b2 but before a second vaccination (‘≥21 d after first dose, no second vaccination BNT162b2’);
vii. Visits 21 d or more after a first vaccination with ChAdOx1 but before a second vaccination (‘≥21 d after first dose, no second vaccination ChAdOx1’);
viii. Visits 21 d or more after a first vaccination with mRNA-1273 but before a second vaccination (‘≥21 d after first dose, no second vaccination mRNA-1273’);
ix. Visits 0–13 d after a second vaccination with BNT162b2 (‘Second dose 0–13 d ago BNT162b2’);
x. Visits 0–13 d after a second vaccination with ChAdOx1 (‘Second dose 0–13 d ago ChAdOx1’);
xi. Visits ≥14 d after second vaccination with BNT162b2 (‘≥14 d after second dose BNT162b2’);
xii. Visits ≥14 d after second vaccination with ChAdOx1 (‘≥14 d after second dose ChAdOx1’);
xiii. Visits from participants who had not yet been vaccinated but were previously PCR/antibody positive in the study or had a positive swab test in the national testing program based on the definition of positive episodes above (‘Not vaccinated, previously positive’).

We chose these vaccination status categories empirically based on previous findings^13^. Exposure group ii (Not vaccinated, not previously positive, 1–21 d before vaccination) was included because there is inevitably a degree of transient reverse causality where vaccination appointments have to be rescheduled if someone tests positive in the weeks before the scheduled visit. Prior infection status was based on multiple sources, including previous PCR-positive episodes in the study, positive tests from the national testing program in England, positive S-antibody measurements before vaccination and N-antibody measurements. All participants were swabbed from enrollment and onwards, allowing assessment of prior infection status via this route. Everyone living in England (83% of the study population) was eligible to get tested via the national testing program if they experienced symptoms or this was required for workplace or school attendance. In total, 19% of participants had an S-antibody measurement before vaccination, and 32% of participants had at least two N-antibody measurements. We defined prior positivity as having either a previous PCR-positive episode or a positive S-antibody measurement more than 90 d before the visit or two consecutive positive N-antibody measurements more than 42 d before the visit. The choice of 90 d and 42 d was arbitrary but designed to exclude ongoing infections acquired previously being misattributed to current visits. Visits from vaccinated individuals (groups (iii)–(xii)) were defined irrespective of previous positivity (Supplementary Table 2) to reflect the effect of vaccination as being implemented in the UK (without regard to prior infection). However, in sensitivity analysis, we analyzed the effect of vaccination by prior infection status. Visits from the same participant were classified in different groups depending on their status at each visit.

### Outcomes

Analysis was based on visits, because these occur independently of symptoms and are, therefore, unbiased. Only the first test-positive visit in each new PCR-positive infection episode starting after 1 December 2020 was used, dropping all subsequent visits in the same infection episode and all negative visits before the first time that a participant could be considered ‘at risk’ for a subsequent new positive episode (as defined above), to avoid misattributing ongoing PCR positivity to visit characteristics and immortal time bias, respectively. Primary analysis included all new PCR-positive episodes. Secondary analyses considered infection severity, by classifying positives by Ct value (<30 or ≥30) and self-reported symptoms. The threshold Ct value of 30 is somewhat arbitrary but corresponds to ~150 copies per ml^29^ and is consistently used in the UK for many purposes, including algorithms for review of low-level positives at the laboratories where the PCR tests were performed and a threshold for attempting whole genome sequencing. For each positive test, a single Ct was calculated as the arithmetic mean across detected genes (Spearman correlation >0.98), and then the minimum value was taken across positives in the infection episode to reflect the greatest measured viral burden within an episode. To allow for pre-symptomatic positives being identified in the survey, any self-reported symptoms at any visit within 0–35 d after the index positive in each infection episode were included (questions elicit symptoms in the last 7 d at each visit). Finally, positive infection episodes were classified as triple positive (ORF1ab + N + S or ORF1ab + S or N + S at least once across the episode; B.1.617.2 compatible), positive only for ORF1ab + N across the episode and never S-positive (B.1.1.7 compatible, because B.1.1.7 has deletions in the S gene leading to S gene target failure) or always positive only on a single gene. As S-gene target failure might also occur in high-Ct samples, the main analysis considered two periods of time when B.1.1.7 dominated (1 December 2020 to 16 May 2021) and when B.1.617.2 dominated (17 May 2021 onwards) (Extended Data Fig. 1), further dividing analysis of Ct values at 14 June 2021.

### Confounders

The following potential confounders were adjusted for in all models for VE as potential risk factors for acquiring SARS-CoV-2 infection (without variable selection): geographic area and age in years (see below), sex, ethnicity (white versus non-white as small numbers), index of multiple deprivation (percentile, calculated separately for each country in the UK; https://www.gov.uk/government/statistics/english-indices-of-deprivation-2019; https://gov.wales/welsh-index-multiple-deprivation-full-index-update-ranks-2019; https://www.gov.scot/collections/scottish-index-of-multiple-deprivation-2020/; https://www.nisra.gov.uk/statistics/deprivation/northern-ireland-multiple-deprivation-measure-2017-nimdm2017), working in a care home, having a patient-facing role in health or social care, presence of long-term health conditions, household size, multigenerational household, rural–urban classification (https://www.nisra.gov.uk/support/geography/urban-rural-classification; https://www.gov.uk/government/collections/rural-urban-classification; https://www.ons.gov.uk/methodology/geography/geographicalproducts/ruralurbanclassifications; https://www.gov.scot/publications/scottish-government-urban-rural-classification-2016/pages/2/), direct or indirect contact with a hospital or care home, smoking status and visit frequency. Details are shown in Supplementary Table 1.

### Statistical analysis

Associations among the different exposure groups and outcome (first positive test in an infection episode versus test negative) were evaluated with generalized linear models with a logit link. Robust standard errors were used to account for multiple visits per participant. To adjust for substantial confounding by calendar time and age, with non-linear effects of age, which are also different by region, we included both as restricted cubic splines and interactions between these splines and region/country (regions for England and country for Northern Ireland, Scotland and Wales). Furthermore, given previous observations of different positivity rates by age over time^12^, we added a tensor spline to model the interaction between age and calendar time with the restriction that the interaction is not doubly non-linear^34^. The primary analysis considered effect modification of each vaccine exposure group by time period (before 17 May 2021 (B.1.1.7 dominant) or after 17 May 2021 (B.1.617.2 dominant)) in those aged ≥18 years. Secondary analyses considered variation over time from second vaccination (linear on the log-odds scale, truncating at the 95th percentile of observed days from second vaccination separately for each vaccine) and effect modification by long-term health conditions, dosing interval and prior infection status in the B.1.617.2-dominant period only in those aged 18–64 years. Pairwise comparisons of the exposure groups were performed unadjusted. Analysis was based on complete cases (>99% of observations).

For all infections, comparisons of Ct values by vaccine exposure groups used quantile (median) regression adjusted for age and sex. Associations between factors and Ct values in ‘breakthrough’ infections occurring ≥14 d after second vaccinations were assessed using mixture normal linear regression models with two component subpopulations (Bayesian Information Criterion 499.4 lower than single population). For these analyses of Ct values, we conducted backwards elimination (exit *P* = 0.05) for associations between factors and the latent class probabilities and separately with the Ct values in each subpopulation for the 12 variables shown in Supplementary Table 7. We included interactions with vaccine in either part of the model type where these had interaction *P* < 0.05. We considered three knot-restricted natural cubic splines in continuous factors (calendar date of positive, age, interval between first and second vaccination and time since second vaccination) (knots at the 10th, 50th and 95th percentiles) if there was evidence of non-linearity at *P* < 0.01. To reduce the influence of outliers, we truncated the interval between first and second vaccination at 3 and 14 weeks and the time from second vaccination at the 95th percentile (118 d, 3.9 months).

### Reporting Summary

Further information on research design is available in the Nature Research Reporting Summary linked to this article.

## Online content

Any methods, additional references, Nature Research reporting summaries, source data, extended data, supplementary information, acknowledgements, peer review information; details of author contributions and competing interests; and statements of data and code availability are available at 10.1038/s41591-021-01548-7.

## Supplementary information

Supplementary Information Supplementary Tables 1–8

Reporting Summary

## Data Availability

Data are still being collected for the COVID-19 Infection Survey. De-identified study data are available for access by accredited researchers in the ONS Secure Research Service (SRS) for accredited research purposes under part 5, chapter 5 of the Digital Economy Act 2017. For further information about accreditation, contact research.support@ons.gov.uk or visit the SRS website.

## Code availability

All statistical analyses of VE were performed using standard functions in the following R packages: ggplot2 (version 3.3.2), rms (version 6.0-1), dplyr (version 1.0.2), emmeans (version 1.5.1), haven (version 2.3.1), sandwich (version 3.0-0), ggeffects (version 1.0.1), broom (version 0.7.2), multcomp (version 1.4–14) and Epi (version 2.44). Analyses of Ct values were performed using qreg and fmm in Stata version 16.1. Code used for data analysis is available upon reasonable request.
