# Supplementary material for: Effect of Delta variant on viral burden and vaccine effectiveness against new SARS-CoV-2 infections in the UK
Source: Nat Med. 2021 Oct 14;27(12):2127–35. doi: 10.1038/s41591-021-01548-7 (PMC8674129; doi:10.1038/s41591-021-01548-7)
Supplement: Supplementary file 1 — Supplementary Tables 1–8 [file 41591_2021_1548_MOESM1_ESM.pdf]

nature portfolio
https://doi.org/10.1038/s41591-021-01548-7

Supplementary information

Effect of Delta variant on viral burden and vaccine effectiveness against new SARS-CoV-2 infections in the UK

In the format provided by the authors and unedited

Supplementary Table 1: Characteristics of visits included in the analyses

Characteristic	1 Dec 2020-16 May 2021 (B.1.1.7-dominant)<br><br>≥18 years old<br><br>Total, n (%) or median (IQR)	17 May 2021 – 2 August (B.1.617.2-dominant)<br><br>≥18 years old<br><br>Total, n (%) or median (IQR)	17 May 2021 – 2 August (B.1.617.2-dominant)<br><br>18-34 years<br><br>Total, n (%) or median (IQR)	17 May 2021 – 2 August (B.1.617.2-dominant)<br><br>35-64 years<br><br>Total, n (%) or median (IQR)
**Female**	1,383,521 (53.6)	439,890 (54.2)	62,747 (54.6)	234,514 (55.8)
**White ethnicity**	2,418,424 (93.7)	756,724 (93.2)	101,134 (87.9)	385,946 (91.9)
**Age**	56 (41,68)	57 (42,69)	28 (23,32)	52 (44,58)
**Region / country**				
London	451,476 (17.5)	140,057 (17.3)	29,154 (25.3)	77,441 (18.4)
North West England	290,953 (11.3)	83,529 (10.3)	11,435 (9.9)	42,585 (10.1)
North East England	95,547 (3.7)	30,158 (3.7)	3,892 (3.4)	15,091 (3.6)
Yorkshire	204,631 (7.9)	69,546 (8.6)	9,105 (7.9)	36,058 (8.6)
West Midlands	187,801 (7.3)	62,202 (7.7)	8,843 (7.7)	31,657 (7.5)
East Midlands	158,924 (6.2)	50,893 (6.3)	6,943 (6.0)	26,228 (6.2)
South East England	322,740 (12.5)	106,483 (13.1)	12,721 (11.1)	55,197 (13.1)
South West England	199,878 (7.7)	56,290 (6.9)	5,966 (5.2)	27,392 (6.5)
East England	259,811 (10.1)	67,086 (8.3)	7,974 (6.9)	34,334 (8.2)
Northern Ireland	73,296 (2.8)	22,399 (2.8)	3,146 (2.7)	12,158 (2.9)
Scotland	211,833 (8.2)	73,518 (9.1)	9,841 (8.6)	38,064 (9.1)
Wales	123,131 (4.8)	49,463 (6.1)	6,003 (5.2)	23,914 (5.7)
**Household size**				
1	491,866 (19.1)	168,423 (20.8)	12,886 (11.2)	74,749 (17.8)
2	1,246,647 (48.3)	388,494 (47.9)	46,117 (40.1)	166,359 (39.6)
3	397,242 (15.4)	122,254 (15.1)	27,504 (23.9)	80,720 (19.2)
4	315,769 (12.2)	95,518 (11.8)	18,769 (16.3)	73,227 (17.4)
5+	128,497 (5.0)	36,935 (4.6)	9,747 (8.5)	25,064 (6.0)
**Multigenerational household**	111,165 (4.3)	35,389 (4.4)	4,825 (4.2)	28,576 (6.8)
**Rural-urban classification**				
Major urban	915,065 (35.5)	292,442 (36)	53,528 (46.5)	156,941 (37.4)
Urban city/town	1,095,963 (42.5)	344,593 (42.5)	45.766 (39.8)	177,297 (42.4)
Rural town	279,076 (10.8)	86,148 (10.6)	8,470 (7.4)	42,239 (10.1)
Rural village	289,917 (11.2)	88,441 (10.9)	7,259 (6.3)	43,642 (10.4)
**Deprivation centile (1 = most deprived, 10 = least deprived)**	6 (3,8)	6 (3,8)	5 (3,7)	6 (3,8)
**Ever reported to be a care home worker**	30,050 (1.2)	9,266 (1.1)	1,742 (1.5)	6,739 (1.6)
**Ever reported to be a person-facing healthcare worker**	66,238 (2.6)	20,850 (2.6)	4,134 (3.6)	15,219 (3.6)
**Ever reported to be person-facing social care worker**	29,691 (1.2)	10,261 (1.3)	1,705 (1.5)	7,687 (1.8)

**Ever reported to have a long-term health condition**	722,819 (28.0)	231,416 (28.5)	20,099 (17.5)	101,659 (24.2)
**Days since previous visit**				
>14 days	1.772,532 (68.7)	707,099 (87.1)	97,880 (85.1)	367,294 (87.4)
<= 14 days	662,234 (25.7)	78,135 (9.6)	12,671 (11.0)	39,603 (9.4)
Enrolment	145,255 (5.6)	26,390 (3.3)	4,472 (3.9)	13,222 (3.1)
**Smoking status**				
Non-smoker	2,325,890 (90.2)	731,654 (90.1)	101,670 (88.4)	370,618 (88.2)
Tobacco smoker	195,301 (7.6)	61,758 (7.6)	9,915 (8.6)	37,509 (8.9)
Only vape	58,830 (2.3)	18,212 (2.2)	3,438 (3.0)	11,992 (2.9)
**Contact with hospital in last 28 days**				
Missing (n)	34,645	2,607	369	1,320
No	1,995,000 (78.4)	604,317 (74.7)	84,146 (73.4)	312,112 (74.5)
Yes, I have	347,145 (13.6)	138,498 (17.1)	17,817 (15.5)	71,921 (17.2)
No, but someone in household has	203,231 (8.0)	66,202 (8.2)	12,691 (11.1)	34,766 (8.3)
**Contact with care home in last 28 days**				
Missing (n)	42,063	3,628	527	1,821
No	2,452,588 (96.6)	766,973 (94.9)	108,837 (95.1)	394,263 (94.3)
Yes, I have	49,649 (2.0)	26,407 (3.3)	2,722 (2.4)	15,791 (3.8)
No, but someone in household has	35,721 (1.4)	14,616 (1.8)	2,937 (2.6)	8,244 (2.0)

Note: analysis is based on visits rather than participants and restricted to those either being unvaccinated or vaccinated with ChAdOx1, BNT162b2 or mRNA-1273: factors above and vaccination exposure (detailed in **Supplementary Table 2**) could change over time

Supplementary Table 2: Vaccination and previous infection status for visits included in analysis (18 years and older)

	1 Dec 2020 – 16 May 2021 (B.1.1.7-dominant)		17 May 2021 – 2 August (B.1.617.2-dominant)	
**Vaccination and previous infection status**	**No prior study or national testing programme swab-positive and no antibody positive<br>Number of visits (row %) [number of positives]**	**Prior study or national testing programme swab-positive or antibody positive<br>Number of visits (row %) [number of positives]**	**No prior study or national testing programme swab-positive and no antibody positive<br>Number of visits (row %) [number of positives]**	**Prior study or national testing programme swab-positive or antibody positive<br>Number of visits (row %) [number of positives]**
Not vaccinated, no prior positive, >21 days before vaccination	1,561,154 (100%) [14,440]	0 (0%) [0]	27,135 (100%) [351]	0 (0%) [0]
Not vaccinated, no prior positive, 1-21 days before vaccination	220,749 (100%) [665]	0 (0%) [0]	16,754 (100%) [51]	0 (0%) [0]
Vaccinated 0-20 days ago, ChAdOx1	127,572 (95%) [293]	4,169 (5%) [11]	4,435 (92%) [4]	387 (8%) [0]
Vaccinated 0-20 days ago, BNT162b2	70,837 (98%) [419]	1,562 (2%) [6]	23,387 (90%) [175]	2,496 (10%) [10]
Vaccinated 0-20 days ago, mRNA-1273	NA	NA	2,941 (91%) [19]	275 (9%) [1]
≥ 21 days after 1st dose, no second dose ChAdOx1	236,306 (95%) [227]	11,390 (5%) [11]	64,232 (91%) [194]	6,377 (9%) [20]
≥ 21 days after 1st dose, no second dose, BNT162b2	141,968 (96%) [278]	5,259 (4%) [11]	38,505 (91%) [410]	3,998 (9%) [12]
≥ 21 days after 1st dose, no second dose, mRNA-1273	NA	NA	5,710 (91%) [30]	534 (9%) [2]
0-13 days after second dose ChAdOx1	32,239 (95%) [14]	1,657 (5%) [2]	44,879 (91%) [91]	4,429 (9%) [1]
0-13 days after second dose, BNT162b2	34,406 (96%) [28]	1,542 (4%) [0]	11,146 (90%) [40]	1,184 (10%) [1]

≥ 14 days after second dose ChAdOx1	30,178 (95%) [9]	1,562 (5%) [1]	303,511 (92%) [1,175]	27,166 (8%) [61]
≥ 14 days after second dose, BNT162b2	70,058 (96%) [39]	3,231 (4%) [4]	199,411 (93%) [422]	16,166 (7%) [25]
Not vaccinated, prior positive	0 (0%) [0]	24,256 (100%) [80]	0 (0%) [0]	5,753 (100%) [25]

Note: analysis is based on visits rather than participants: factors (**Table S1**) and vaccination exposure could change over time. See methods for definition of PCR-positive episodes of infection and prior positivity. Cells with zeros only are by definition. NA indicates to few visits to estimate effectiveness.

Supplementary Table 3. Vaccine effectiveness (VE) (95% CI) for single mRNA-1273 dose in those 18 to 64 years in the B.1.617.2-dominant period.

**Vaccine effectiveness (95% CI)**	**mRNA-1273: one dose ≥21 days**
All infections	75% (64% - 83%)
Ct<30	77% (67% - 84%)
Self-reported symptoms	77% (66% - 84%)
Ct≥30	67% (53% - 77%)
No self-reported symptoms	70% (57% - 79%)

Note: see **Table 2** for VE for other vaccines.

Supplementary Table 4. Comparisons between vaccines and with previous infection (unadjusted heterogeneity p-values) in those aged 18 years and older in B.1.1.7-dominant and B.1.617.2-dominant periods.

	BNT162b2 vs ChAdOx1: one dose ≥21 days	BNT162b2 vs ChAdOx1: second dose 0-13 days ago	BNT162b2 vs ChAdOx1: second dose ≥14 days	BNT162b2: second dose ≥14 days vs not vaccinated, previously positive*	ChAdOx1: second dose ≥14 days vs not vaccinated, previously positive*	BNT162b2: second dose ≥14 days vs one dose ≥21 days	ChAdOx1: second dose ≥14 days vs one dose ≥21 days
**All infections**							
1 Dec 2020 – 16 May 2021 (B.1.1.7)	0.34	0.54	0.85			0.0007	0.12
17 May 2021- (B.1.617.2)	0.01	0.01	<0.0001	0.12	0.38	<0.0001	<0.0001
**Ct<30**							
1 Dec 2020 – 16 May 2021 (B.1.1.7)	0.08	0.44	0.03			<0.0001	0.10
17 May 2021- (B.1.617.2)	0.003	0.02	<0.0001	0.09	0.15	<0.0001	<0.0001
**Self-reported symptoms**							
1 Dec 2020 – 16 May 2021 (B.1.1.7)	0.93	0.04	0.52			<0.0001	<0.0001
17 May 2021- (B.1.617.2)	0.0001	<0.0001	<0.0001	0.59	0.01	<0.0001	<0.0001

Note: Calendar time split into two epochs when the majority of cases detected in the survey were ORF1ab+N positive (B.1.1.7-compatible) and then when triple positives became dominant (B.1.617.2-compatible) (**Extended Data Fig. 1**). P-values were obtained using two-sided Wald test without adjustment for multiple comparisons.

Supplementary Table 5. Comparisons between vaccines and with previous infection (unadjusted heterogeneity p-values) in those aged 18 to 64 years in the B.1.617.2-dominant period

	BNT162b2 vs ChAdOx1: one dose ≥21 days	BNT162b2 vs ChAdOx1: second dose 0-13 days ago	BNT162b2 vs ChAdOx1: second dose ≥14 days	BNT162b2: second dose ≥14 days vs not vaccinated, previously positive*	ChAdOx1: second dose ≥14 days vs not vaccinated, previously positive*	BNT162b2: second dose ≥14 days vs one dose ≥21 days	ChAdOx1: second dose ≥14 days vs one dose ≥21 days
All infections (Figure 1A)	0.001	0.006	<0.0001	0.04	0.33	<0.0001	<0.0001
Ct<30 (Figure 1B)	0.0003	0.02	<0.0001	0.02	0.12	<0.0001	<0.0001
Self-reported symptoms (Figure 1C)	<0.0001	<0.0001	<0.0001	0.33	0.01	<0.0001	<0.0001
Ct≥30	0.03	0.0002	<0.0001	0.07	0.81	<0.0001	<0.0001
No self-reported symptoms	0.24	0.27	<0.0001	0.003	0.49	<0.0001	0.05

Note: Calendar time split into two epochs when the majority of cases detected in the survey were ORF1ab+N positive (B.1.1.7-compatible) and then when triple positives became dominant (B.1.617.2-compatible) (**Extended Data Fig. 1**).

Supplementary Table 6: Vaccine effectiveness by subgroups during the B.1.617.2-dominant period in those aged 18-64 years

	VE against all infections			VE against infections with Ct<30			VE against infections with reported symptoms		
**By evidence of prior infection**	**No evidence of prior infection**	**Evidence of prior infection**	**Hetero-geneity p-value**	**No evidence of prior infection**	**Evidence of prior infection**	**Hetero-geneity p-value**	**No evidence of prior infection**	**Evidence of prior infection**	**Hetero-geneity p-value**
≥21 days after 1st dose, no second vaccination BNT162b2	57% (50-63%)	91% (84-95%)	**<0.0001**	62% (54-68%)	96% (88-98%)	**<0.0001**	58% (50-65%)	94% (87-98%)	**<0.0001**
≥21 days after 1st dose, no second vaccination ChAdOx1	43% (30-54%)	53% (24-71%)	0.45	45% (30-57%)	78% (49-90%)	0.03	34% (16-48%)	83% (55-94%)	**0.007**
≥21 days after 1st dose, no second vaccination mRNA-1273	76% (64-83%)	86% (44-97%)	0.43	76% (62-85%)	100% (100-100%)	**<0.0001**	76% (62-85%)	100% (100-100%)	**<0.0001**
14 days after 2nd dose BNT162b2	85% (79-90%)	93% (87-96%)	**0.006**	92% (87-95%)	98% (94-99%)	**0.004**	93% (89-97%)	99% (96-100%)	**0.002**
14 days after 2nd dose ChAdOx1	68% (61-73%)	88% (83-92%)	**<0.0001**	69% (61-75%)	92% (87-95%)	**<0.0001**	72% (64-78%)	94% (89-97%)	**<0.0001**
**By age**	**Aged 18-34 years old**	**Aged 35-64 years old**	**Hetero-geneity p-value**	**Aged 18-34 years old**	**Aged 35-64 years old**	**Hetero-geneity p-value**	**Aged 18-34 years old**	**Aged 35-64 years old**	**Hetero-geneity p-value**
≥21 days after 1st dose, no second vaccination BNT162b2	64% (57-70%)	36% (14-53%)	**0.0009**	68% (60-74%)	46% (22-62%)	**0.01**	66% (58-72%)	32% (0-53%)	0.002

	VE against all infections			VE against infections with Ct<30			VE against infections with reported symptoms		
≥21 days after 1st dose, no second vaccination ChAdOx1	43% (11-64%)	18% (-9-39%)	0.18	57% (21-77%)	22% (-9-45%)	0.10	37% (-7% -63%)	0% (-43-31%)	0.16
≥21 days after 1st dose, no second vaccination mRNA-1273	82% (70-89%)	56% (23-75%)	0.02	81% (66-90%)	64% (27-83%)	0.18	85% (72-92%)	48% (-1-73%)	**0.008**
14 days after 2nd dose BNT162b2	90% (85-93%)	77% (65-85%)	**0.0001**	95% (91-97%)	88% (79-93%)	**0.002**	96% (93-98%)	88% (78-94%)	**<0.0001**
14 days after 2nd dose ChAdOx1	73% (65-80%)	54% (40-65%)	**0.002**	74% (64-81%)	57% (41-69%)	0.02	76% (67-83%)	57% (39-70%)	**0.007**
**By interval between first and second doses**	**Dosing interval <6 weeks**	**Dosing interval ≥6 weeks**	**Hetero-geneity p-value**	**Dosing interval <6 weeks**	**Dosing interval ≥6 weeks**	**Hetero-geneity p-value**	**Dosing interval <6 weeks**	**Dosing interval ≥6 weeks**	**Hetero-geneity p-value**
14 days after 2nd dose BNT162b2	82% (72-88%)	86% (80-90%)	0.18	90% (83-95%)	93% (88-95%)	0.30	91% (83-95%)	94% (89-96%)	0.13
**By self-reported long-term health conditions**	**No long-term health conditions**	**Long-term health conditions**	**Hetero-geneity p-value**	**No long-term health conditions**	**Long-term health conditions**	**Hetero-geneity p-value**	**No long-term health conditions**	**Long-term health conditions**	**Hetero-geneity p-value**
≥21 days after 1st dose, no second vaccination BNT162b2	58% (50-64%)	62% (39-77%)	0.65	62% (54-69%)	66% (40-80%)	0.76	58% (49-66%)	66% (40-81%)	0.48
≥21 days after 1st dose, no second vaccination ChAdOx1	45% (32-55%)	7% (-48-41%)	0.04	46% (30-58%)	28% (-27-59%)	0.35	38% (19-52%)	5% (-66-46%)	0.18

	VE against all infections			VE against infections with Ct<30			VE against infections with reported symptoms		
≥21 days after 1st dose, no second vaccination mRNA-1273	77% (65-84%)	59% (-17-85%)	0.31	78% (65-87%)	59% (-35-88%)	0.33	80% (66-88%)	44 (-61-81%)	0.09
14 days after 2nd dose BNT162b2	86% (80-90%)	81% (69-89%)	0.23	92% (87-95%)	92% (85-96%)	0.96	94% (89-96%)	92% (84-96%)	0.38
14 days after 2nd dose ChAdOx1	69% (62-74%)	58% (39-71%)	0.10	70% (62-76%)	65% (46-77%)	0.48	73% (65-79%)	64% (44-77%)	0.23

Note: obtained using a model with logit-linear slopes on time since ≥14 days after second ChAdOx1 or BNT162b2 vaccination. The effect estimates in the table relate to the effectiveness on the 14th day after the second dose ('intercept' of the second dose effect). Potential waning of effectiveness as a function of time since ≥14 days after the second vaccination is obtained from the same model by including logit-linear time terms for BNT162b2 and ChAdOx1 that are not varied by subgroup due to insufficient numbers to estimate different slopes by the combination of vaccine type and subgroup effects (potential waning effects shown in **Figure 2**, **Extended Data Fig. 4 & 5**). P-values<0.01 shown in bold (0.01 threshold used given the number of tests performed). P-values were obtained using two-sided Wald test without adjustment for multiple comparison.

Supplementary Table 7: Median (IQR) Ct values in all new PCR-positives

	B.1.1.7-dominant period<br>1 December 2020-16 May 2021				Early B.1.617.2-dominant period<br>17 May-13 June 2021				Late B.1.617.2-dominant period<br>17 May-13 June 2021			
	N	Median	lower 25th percentile	upper 75th percentile	N	Median	lower 25th percentile	upper 75th percentile	N	Median	lower 25th percentile	upper 75th percentile
Not vaccinated	10853	28.7	20.4	32.9	75	21.5	16.4	31.7	326	25.7	19.1	30.8
0-20 days of first dose	733	31.0	22.8	33.7	24	30.0	21.9	34.2	185	25.6	21.6	30.9
≥21 days after first dose or <14 days after 2nd dose	577	31.6	26.6	33.7	110	30.1	18.6	33.7	705	24.7	18.8	31.3
≥14 days after 2nd dose	56	33.3	31.6	34.0	104	32.2	26.0	34.0	1593	25.3	19.1	31.3
Reinfection before vaccination	68	32.8	30.9	34.2	5	30.8	29.5	34.3	20	22.3	16.5	30.3

Supplementary Table 8: Independent associations with Ct values in new PCR-positives ≥14 days after second vaccination 18 years and older

	**Low Ct vs high Ct: odds of class membership**			**Low Ct sub-population: mean Ct**			**High Ct sub-population: mean Ct**			Het low vs high mean
	OR	95% CI	p	Effect	95% CI	p	Effect	95% CI	p	p
Overall mean Ct				**+21.2**	**+18.5,+23.8**	**<0.0001**	**33.0**	**+32.4,+33.5**	**<0.0001**	<0.0001
Per month post 1 Jan 2021 (reference 27 April) 2021	**1.76**	**1.41,2.20**	**<0.0001**	**Fig S5C**		**0.0002**	**Fig S5C**		**0.0004**	
Vaccine: 14 days post second dose										
ChAdOx1	1.00 (ref)		-	0.0 (ref)		-	0.0 (ref)		-	
BNT162b2	**0.33**	**0.16,0.67**	**0.002**	**+4.0**	**+1.4,+6.6**	**0.003**	+0.5	-0.2,+1.2	0.16	0.008
Vaccine: per month later post second dose										
ChAdOx1	0.97	0.79,1.19	0.78	-0.2	-0.8,+0.3	0.45	-0.1	-0.3,+0.2	0.66	0.58
BNT162b2	**1.43**	**1.07,1.91**	**0.01**	**-1.6**	**-2.5,-0.6**	**0.002**	-0.0	-0.3,+0.3	0.82	0.01
Previously PCR/antibody-positive	**0.35**	**0.21,0.57**	**<0.0001**			*0.58*			*0.10*	
Report having a long-term health condition	**0.69**	**0.53,0.90**	**0.006**			*0.78*			*0.52*	
Sex: Male			*0.34*	0.0 (ref)		-	0.0 (ref)		-	
Female			-	+0.0	-0.7,+0.7	0.98	**+0.3**	**+0.0,+0.6**	**0.04**	0.39
Age			*0.19*			*0.24*			*0.64*	
Ethnicity (white vs non-white)			*0.96*			*0.65*			*0.85*	
Patient facing healthcare worker			*0.63*			*0.90*			*0.28*	
Deprivation percentile			*0.60*			*0.94*			*0.91*	
Interval between first and second dose			*0.83*			*0.64*			*0.16*	

Note: where effect size not shown (gray cells), p-values in italics show the additional effect of adding this term into the model for either class membership or for and effect on mean Ct in the two sub-populations. Test for interaction between effect of vaccine type and time since second dose: p=0.02 for membership of high vs low Ct sub-population, p=0.01 for low mean Ct, p=0.89 for high mean Ct. Excluding 17 positives occurring ≥14 days after second vaccination but not reporting either two ChAdOx1 or two BNT162b2 doses. There was no evidence of interaction between vaccine type and age for class probability (p=0.09) or mean Ct (p>0.1); nor was there evidence of interaction between vaccine type and date for class probability (p=0.69) or mean Ct (p>0.4). P-values were obtained using two-sided Wald test without adjustment for multiple comparisons.
