# Supplementary material for: Effect of Delta variant on viral burden and vaccine effectiveness against new SARS-CoV-2 infections in the UK
Source: Nat Med. 2021 Oct 14;27(12):2127–35. doi: 10.1038/s41591-021-01548-7 (PMC8674129; doi:10.1038/s41591-021-01548-7)
Supplement: Supplementary file 2 — Reporting Summary [file 41591_2021_1548_MOESM2_ESM.pdf]

nature research

Corresponding author(s): Koen Pouwels

Last updated by author(s): Sep 17, 2021

Reporting Summary

Nature Research wishes to improve the reproducibility of the work that we publish. This form provides structure for consistency and transparency in reporting. For further information on Nature Research policies, see our Editorial Policies and the Editorial Policy Checklist.

Statistics

For all statistical analyses, confirm that the following items are present in the figure legend, table legend, main text, or Methods section.

n/a	Confirmed	
☐	☒	The exact sample size (*n*) for each experimental group/condition, given as a discrete number and unit of measurement
☐	☒	A statement on whether measurements were taken from distinct samples or whether the same sample was measured repeatedly
☐	☒	The statistical test(s) used AND whether they are one- or two-sided<br>*Only common tests should be described solely by name; describe more complex techniques in the Methods section.*
☐	☒	A description of all covariates tested
☐	☒	A description of any assumptions or corrections, such as tests of normality and adjustment for multiple comparisons
☐	☒	A full description of the statistical parameters including central tendency (e.g. means) or other basic estimates (e.g. regression coefficient) AND variation (e.g. standard deviation) or associated estimates of uncertainty (e.g. confidence intervals)
☐	☒	For null hypothesis testing, the test statistic (e.g. *F*, *t*, *r*) with confidence intervals, effect sizes, degrees of freedom and *P* value noted<br>*Give P values as exact values whenever suitable.*
☒	☐	For Bayesian analysis, information on the choice of priors and Markov chain Monte Carlo settings
☒	☐	For hierarchical and complex designs, identification of the appropriate level for tests and full reporting of outcomes
☐	☒	Estimates of effect sizes (e.g. Cohen's *d*, Pearson's *r*), indicating how they were calculated

Our web collection on statistics for biologists contains articles on many of the points above.

Software and code

Policy information about availability of computer code

Data collection	De-identified data were accessed through the Office for National Statistics (ONS) Secure Research Service (SRS). The data available in SRS were prepared for data analysis using Stata MP 16.1.
Data analysis	All statistical analyses of vaccine effectiveness were performed using standard functions in the following R packages: ggplot2 (version 3.3.2), rms (version 6.0-1), dplyr (version 1.0.2), emmeans (version 1.5.1), haven (version 2.3.1), sandwich (version 3.0-0), ggeffects (version 1.0.1), broom (version 0.7.2), multcomp (version 1.4-14), and Epi (version 2.44)). Analyses of Ct values were performed using logit, qreg and fmm in Stata v16.1.Code used for data analysis is available upon request.

For manuscripts utilizing custom algorithms or software that are central to the research but not yet described in published literature, software must be made available to editors and reviewers. We strongly encourage code deposition in a community repository (e.g. GitHub). See the Nature Research guidelines for submitting code & software for further information.

Data

Policy information about availability of data

All manuscripts must include a data availability statement. This statement should provide the following information, where applicable:

- Accession codes, unique identifiers, or web links for publicly available datasets
- A list of figures that have associated raw data
- A description of any restrictions on data availability

Data are still being collected for the COVID-19 Infection Survey. De-identified study data are available for access by accredited researchers in the ONS Secure Research Service (SRS) for accredited research purposed under part 5, chapter 5 of the Digital Economy Act 2017. For further information about accreditation, contact Research.Support@ons.gov.uk or visit the SRS website.
Individuals can apply to be an accredited researcher using the short form on https://researchaccreditationservice.ons.gov.uk/ons/ONS_registration.ofml.

Accreditation requires completion of a short free course on accessing the SRS. To request access to data in the SRS, researchers must submit a research project application for accreditation in the Research Accreditation Service (RAS). Research project applications are considered by the project team and the Research Accreditation Panel (RAP) established by the UK Statistics Authority. Project application example guidance and an exemplar of a research project application are available. A complete record of accredited researchers and their projects is published on the UK Statistics Authority website to ensure transparency of access to research data.

Field-specific reporting

Please select the one below that is the best fit for your research. If you are not sure, read the appropriate sections before making your selection.

☒ Life sciences ☐ Behavioural & social sciences ☐ Ecological, evolutionary & environmental sciences

For a reference copy of the document with all sections, see nature.com/documents/nr-reporting-summary-flat.pdf

Life sciences study design

All studies must disclose on these points even when the disclosure is negative.

Sample size

During the Alpha-dominant period from 1 December 2020 to 16 May 2021 (Figure S1), nose and throat RT-PCR results were obtained from 384,543 individuals aged 18 years or older (221,909 households) at 2,580,021 visits (median [IQR] 7 [6-8]), of which 16,538 (0.6%) were the first PCR-positive in a new infection episode. During the Delta-dominant period from 17 May to 1 August 2021, results were obtained from 358,983 individuals (213,825 households) at 811,624 visits (median [IQR] 2 [2-3]), 3,123 (0.4%) being the first PCR-positive.

No sample size calculation was performed for this particular analysis. A recent paper explained why power calculations for observational studies using existing databases trying to address a causal question are not necessary: If a question is important enough there can't be an excuse to do nothing' [1].
1. Hernan MA. Causal analyses of existing databases: no power calculations required. J Clin Epidemiol 2021.

Data exclusions

This analysis included participants aged 18 years or over, and all visits with positive or negative swab results from 1 December 2020 to 1 August 2021.

Replication

Statistical analyses were successfully replicated by the same individual twice. No experiments other than statistical analyses were performed.

Randomization

The following potential confounders were adjusted for in all models as potential risk factors for acquiring SARS-CoV-2 infection: geographic area and age in years (see below), sex, ethnicity (white versus non-white as small numbers), index of multiple deprivation (percentile, calculated separately for each country in the UK), working in a care-home, having a patient-facing role in health or social care, presence of long-term health conditions, household size, multigenerational household, rural-urban classification, direct or indirect contact with a hospital or care-home, smoking status, and visit frequency.

Blinding

Since we compared multiple exposure categories to the same reference and also included splines modeling time since vaccination blinding was not feasible in this observational study.

Reporting for specific materials, systems and methods

We require information from authors about some types of materials, experimental systems and methods used in many studies. Here, indicate whether each material, system or method listed is relevant to your study. If you are not sure if a list item applies to your research, read the appropriate section before selecting a response.

Materials & experimental systems

n/a	Involved in the study	
☒	☐	Antibodies
☒	☐	Eukaryotic cell lines
☒	☐	Palaeontology and archaeology
☒	☐	Animals and other organisms
☐	☒	Human research participants
☐	☒	Clinical data
☒	☐	Dual use research of concern

Methods

n/a	Involved in the study	
☒	☐	ChIP-seq
☒	☐	Flow cytometry
☒	☐	MRI-based neuroimaging

Human research participants

Policy information about studies involving human research participants

Population characteristics

Private households are randomly selected on a continuous basis from address lists and previous surveys to provide a representative sample across the UK. Characteristics at included visits are shown in Supplementary Table 1.

Recruitment	The ONS COVID-19 Infection Survey is a large household survey with longitudinal follow-up (ISRCTN21086382; https://www.ndm.ox.ac.uk/covid-19/covid-19-infection-survey/protocol-and-information-sheets). The study received ethical approval from the South Central Berkshire B Research Ethics Committee (20/SC/0195). Private households are randomly selected on a continuous basis from address lists and previous surveys to provide a representative sample across the United Kingdom. For the current analysis, following verbal agreement to participate, a study worker visited each selected household to take written informed consent for individuals aged 2 years and over. Parents or carers provided consent for those aged 2–15 years; those aged 10–15 years also provided written assent. For the current analysis, we only included individuals aged 18 years and over.<br>While certain factors might drive non-response to invitations to participate, adjustment for covariates that may influence selection into the sample ensures that estimates of relative effects are not biased by factors that both influence selection into the sample and the risk of the outcome (model-based inference). Factors that were included in the model included: geographic area and age in years, sex, ethnicity (white vs non-white as small numbers), index of multiple deprivation (percentile, calculated separately for each country in the UK), working in a care home, having a patient-facing role in health or social care, presence of long-term health conditions, household size, multi-generational household, rural-urban classification, direct or indirect contact with a hospital or care-home, smoking status, and visit frequency. We cannot exclude the possibility that other unmeasured factors that could influence self-selection into the survey and are not strongly associated with factors already included in the model could bias the results.
Ethics oversight	The study received ethical approval from the South Central Berkshire B Research Ethics Committee (20/SC/0195).

Note that full information on the approval of the study protocol must also be provided in the manuscript.

Clinical data

Policy information about clinical studies

All manuscripts should comply with the ICMJE guidelines for publication of clinical research and a completed CONSORT checklist must be included with all submissions.

Clinical trial registration	ISRCTN21086382
Study protocol	https://www.ndm.ox.ac.uk/covid-19/covid-19-infection-survey/protocol-and-information-sheets
Data collection	The Office for National Statistics (ONS) COVID-19 Infection Survey (CIS) is a large household survey with longitudinal follow-up (ISRCTN21086382, https://www.ndm.ox.ac.uk/covid-19/covid-19-infection-survey/protocol-and-information-sheets). The study received ethical approval from the South Central Berkshire B Research Ethics Committee (20/SC/0195). Private households are randomly selected on a continuous basis from address lists and previous surveys to provide a representative sample across the UK. Following verbal agreement to participate, a study worker visited each selected household to take written informed consent for individuals aged 2 years and over. Parents or carers provided consent for those aged 2-15 years; those aged 10-15 years also provided written assent. For the current analysis we only included individuals aged 18 years and over.<br>Individuals were asked about demographics, behaviours, work, and vaccination uptake (https://www.ndm.ox.ac.uk/covid-19/covid-19-infection-survey/case-record-forms). At the first visit, participants were asked for (optional) consent for follow-up visits every week for the next month, then monthly for 12 months from enrolment. At each visit, enrolled household members provided a nose and throat self-swab following instructions from the study worker. From a random 10-20% of households, those 16 years or older were invited to provide blood monthly for antibody testing from enrolment. From April 2021, additional participants were invited to provide blood samples monthly to assess vaccine responses, based on a combination of random selection and prioritisation of those in the study for the longest period (independent of test results). Throughout, participants with a positive swab test and their household members were also invited to provide blood monthly for follow-up visits after this. The first participant was recruited to the survey on 26 April 2020 and data up to 1 August (the most recent data available at the time of the analyses) are included in this particular study.
Outcomes	Analysis was based on visits, since these occur independently of symptoms and are therefore unbiased. Only the first test-positive visit in each new PCR-positive infection episode starting after 1 December 2020 was used, dropping all subsequent visits in the same infection episode and all negative visits before the first time a participant could be considered "at risk" for a subsequent new positive episode (as defined above), to avoid misattributing ongoing PCR-positivity to visit characteristics and immortal time bias respectively. Primary analysis included all new PCR-positive episodes. Secondary analyses considered infection severity, by classifying positives by cycle threshold (Ct) value (<30 or ≥30) and self-reported symptoms. The threshold Ct value of 30 is somewhat arbitrary, but corresponds to ~150 copies/ml, and is consistently used in the UK for many purposes, including algorithms for review of low level positives at the laboratories where the PCR tests were performed and a threshold for attempting whole genome sequencing. For each positive test, a single Ct was calculated as the arithmetic mean across detected genes (Spearman correlation>0.98), then the minimum value was taken across positives in the infection episode to reflect the greatest measured viral burden within an episode. To allow for pre-symptomatic positives being identified in the survey, any self-reported symptoms at any visit within 0 to 35 days after the index positive in each infection episode were included (questions elicit symptoms in the last 7 days at each visit). Finally, positive infection episodes were classified as triple positive (ORF1ab+N+S or ORF1ab+S or N+S at least once across the episode; Delta-compatible), positive only for ORF1ab+N across the episode and never S-positive (Alpha-compatible, since Alpha has deletions in the S gene leading to S gene target failure) or always positive only on a single gene. As S-gene target failure may also occur in high Ct samples, the main analysis considered two periods of time when Alpha dominated (1 December 2020 to 16 May 2021) and when Delta dominated (17 May 2021 onwards) (Figure S1), further dividing analysis of Ct values at 14 June 2021.
